# Significant Efficiency Enhancements in Non‐Y Series Acceptors by the Addition of Outer Side Chains

**DOI:** 10.1002/advs.202414042

**Published:** 2025-01-22

**Authors:** Qiao He, Wisnu Tantyo Hadmojo, Xiantao Hu, Subhrangsu Mukherjee, Maryam Alqurashi, Wejdan Althobaiti, Catherine S. P. De Castro, Byongkyu Lee, Bowen Ding, Joel Luke, Panagiota Kafourou, Zhuping Fei, Andrew J. P. White, Julien Gorenflot, Florian Glöcklhofer, Frédéric Laquai, Harald Ade, Thomas D. Anthopoulos, Martin Heeney

**Affiliations:** ^1^ Department of Chemistry and Centre for Processable Electronics Imperial College London London W12 0BZ UK; ^2^ Division of Physical Sciences and Engineering King Abdullah University of Science and Technology and KAUST Solar Center Thuwal 23955‐6900 Saudi Arabia; ^3^ Department of Physics and Organic and Carbon Electronics Laboratories (ORaCEL) North Carolina State University Raleigh NC 27695 USA; ^4^ Institute of Molecular Plus and Department of Chemistry Tianjin Key Laboratory of Molecular Optoelectronic Science Tianjin University Tianjin 300072 China; ^5^ Institute of Applied Synthetic Chemistry TU Wien Vienna 1060 Austria; ^6^ Department of Chemistry LMU Munich Butenandtstraße 5–13 (E) D‐81377 München Germany; ^7^ Department of Electrical and Electronic Engineering Henry Royce Institute and Photon Science Institute The University of Manchester Manchester M13 9PL UK

**Keywords:** IDTT, organic solar cell, side chain engineering, small molecule acceptor, steric hindrance

## Abstract

Most current highly efficient organic solar cells utilize small molecules like Y6 and its derivatives as electron acceptors in the photoactive layer. In this work, a small molecule acceptor, SC8‐IT4F, is developed through outer side chain engineering on the terminal thiophene of a conjugated 6,12‐dihydro‐dithienoindeno[2,3‐d:2′,3′‐d′]‐s‐indaceno[1,2‐b:5,6‐b′]dithiophene (IDTT) central core. Compared to the reference molecule C8‐IT4F, which lacks outer side chains, SC8‐IT4F displays notable differences in molecule geometry (as shown by simulations), thermal behavior, single‐crystal packing, and film morphology. Blend films of SC8‐IT4F and the polymer donor PM6 exhibit larger carrier mobilities, longer carrier lifetimes, and reduced recombination compared to C8‐IT4F, resulting in improved device performance. Binary photovoltaic devices based on the PM6:SC8‐IT4F films reveal an optimal efficiency over 15%, which is one of the best values for non‐Y type small molecule acceptors (SMAs). The resultant devices also show better thermal and operational stability than the control PM6:L8‐BO devices. SC8‐IT4F and its blend exhibit a higher relative degree of crystallinity and π coherence length, compared to C8‐IT4F samples, beneficial for charge transport and device performance. The results indicate that outer side chain engineering on existing small electron acceptors can be a promising molecular design strategy for further pursuing high‐performance organic solar cells.

## Graphical abstract

A novel small molecule acceptor SC8‐IT4F has been synthesized by introducing outer alkyl chains onto the terminal thiophene of the conjugated IDTT core and investigated for high‐performance organic solar cells. PM6:SC8‐IT4F‐based devices reveal a high power conversion efficiency of 15.13%, which is one of the best values for non‐Y series acceptors.

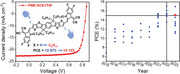

## 1 Introduction

Organic solar cells (OSCs) have emerged as a promising technology for solar energy conversion, yielding impressive power conversion efficiencies (PCEs) surpassing 19% in recent advancements.^[^ ^1^ ^]^ This significant progress in their device performance can be attributed to the identification and utilization of highly efficient photovoltaic materials, primarily consisting of donor and acceptor components.^[^ ^2^ ^]^ Among the various acceptor types, A‐D‐A and A‐DA'D‐A (A,A’ = acceptor, D = donor) structured small molecule acceptors (SMAs) are the most promising candidates.^[^ ^3^ ^]^ In 2015, Zhan et al. reported A‐D‐A‐type SMAs, represented by 3,9‐bis(2‐methylene‐(3‐(1,1‐dicyanomethylene)‐indanone))‐5,5,11,11‐tetrakis(4‐hexylphenyl)‐dithieno[2,3‐*d*:2′,3′‐*d*′]‐s‐indaceno[1,2‐b:5,6‐*b*′]dithiophene (ITIC), 2,2′‐((2Z,2′Z)‐((4,4,9,9‐tetrahexyl‐4,9‐dihydro‐s‐indaceno[1,2‐*b*:5,6‐*b*′]dithiophene‐2,7‐diyl)bis(methanylylidene))bis (3‐oxo‐2,3‐dihydro‐1H‐indene‐2,1‐diylidene))dimalononitrile (IDIC), and 3,9‐bis(2‐methylene‐(3‐(1,1‐dicyanomethylene)‐indanone))‐5,5,11,11‐tetrakis(5‐hexylthienyl)‐dithieno[2,3‐*d*:2′,3′‐*d*′]‐s‐indaceno[1,2‐*b*:5,6‐*b*′]dithiophene (ITIC‐Th).^[^ ^4^ ^]^ Benefitting from the strong intramolecular electron push‐pull effect in the A‐D‐A‐type backbone, these ITIC‐series materials exhibited favorable optoelectronic properties, such as intensive light absorption in the near‐infrared (NIR) region, and appropriate frontier molecular orbital energy levels when matched with wide bandgap (WBG) polymer donors.^[^ ^5^ ^]^ Consequently, the growing set of ITIC‐based materials promoted OSC efficiencies to 11–15%.^[^ ^5^, ^6^ ^]^ Zou et al. and Yang et al. proposed an A‐DA'D‐A‐type skeleton by incorporating an electron‐withdrawing unit into the middle of the central fused ring, resulting in the emergence of the Y‐series SMAs,^[^ ^7^ ^]^ which exhibited increased absorption in the near‐infrared region. More significantly, a 3D charge transport network was formed by π–π stacking of end‐groups and DA'D units, which helped alleviate charge recombination in the active layer.^[^ ^8^ ^]^ Nowadays, Y‐series SMAs are the indispensable component in OSC devices reaching high‐level efficiencies of over 19%.^[^ ^9^ ^]^

SMAs, either ITIC‐ or Y‐ series, consist of a central conjugated core, terminal end groups, and side chains.^[^ ^4^, ^6^, ^10^ ^]^ Subtle molecular modifications on the conjugated core, end groups, and side chains contribute to their diverse chemical properties and morphological aggregation behavior and thus can impact on the performance of SMA‐OSCs. Compared to the other two strategies, side‐chain engineering has the unique advantage of preserving the optoelectronic properties of SMAs while adjusting their solubility and aggregation properties.^[^ ^11^ ^]^ As an example, we previously reported a fully alkylated 6,12‐dihydro‐dithienoindeno [2,3‐*d*:2′,3′‐*d*′]‐s‐indaceno[1,2‐b:5,6‐*b*′]dithiophene (IDTT)‐based SMA (C8‐ITIC) with high crystallinity.^[^ ^12^ ^]^ By comparison of its properties to a common analog with phenylalkyl chains, ITIC, we showed that inner linear alkyl side chains reduced the optical band gap, increased film absorptivity and its propensity to crystallize, leading to a PCE up to 13.2%, higher than that of ITIC. This tactic has also been successful in modifying the high‐performance SMA material 2,2′‐((2Z,2′Z)‐((12,13‐bis(2‐ethylhexyl)‐3,9‐diundecyl‐12,13‐dihydro‐[1,2,5]thiadiazolo[3,4‐*e*]thieno[2″,3″:4′,5′]thieno[2′,3′:4,5]pyrrolo[3,2‐*g*]thieno[2′,3′:4,5]thieno[3,2‐*b*]indole‐2,10‐diyl)bis(methanylylidene))bis(5,6‐difluoro‐3‐oxo‐2,3‐dihydro‐1H‐indene‐2,1‐diylidene))dimalononitrile (Y6), either replacing the inner side chains (on the pyrrole rings) or the outer side chains (on the β position of thiophene).^[^ ^11^, ^13^ ^]^ For example, the Y6‐derivative 2,2′‐((2Z,2′Z)‐((12,13‐bis(2‐ethylhexyl)‐3,9‐(2‐butyloctyl)‐12,13‐dihydro‐[1,2,5]thiadiazolo[3,4‐*e*]thieno[2″,3″:4′,5′]thieno[2′,3′:4,5]pyrrolo[3,2‐*g*]thieno[2′,3′:4,5]thieno[3,2‐*b*]indole‐2,10‐diyl)bis(methanylylidene))bis(5,6‐difluoro‐3‐oxo‐2,3‐dihydro‐1H‐indene‐2,1‐diylidene))dimalononitrile (L8‐BO) with outer branched alkyl side chains instead of linear alkyl chains shows a tighter molecular stacking, weakened intermolecular interactions and raised lowest unoccupied molecular orbital (LUMO) energy level.^[^ ^14^ ^]^ The reduced aggregation causes blue‐shifted light absorption, but the short circuit current density (*J*_SC_) is not sacrificed as the relatively narrowed total spectra coverage is compensated by increased absorption at ≈650–680 nm by the polymer donor and SMA, enabling L8‐BO‐based OSC devices to yield higher overall photovoltaic performance.

**Scheme 1:**
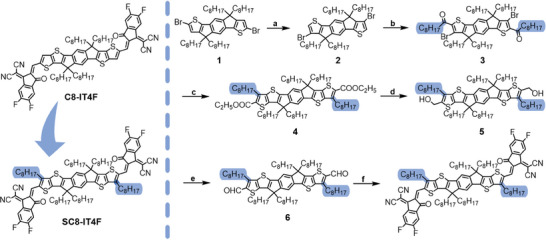
The chemical structures of C8‐IT4F and SC8‐IT4F (left), and the detailed synthetic route to SC8‐IT4F (right): a) LDA/H_2_O, 80%; b) Nonanoyl chloride/AlCl_3_/DCM, 68%; c) Ethyl thioglycolate/K_2_CO_3_/DMF, 76%; d) LiAlH_4_/THF; e) Dess–Martin periodinane/THF, 65%; f) 2‐(5,6‐difluoro‐3‐oxo‐2,3‐dihydro‐1H‐inden‐1‐ylidene)malononitrile/pyridine/CHCl_3_, 78%.

Both the PCEs and device stabilities are key performance concerns in OSC research and their commercial applications.^[^ ^15^ ^]^ However, unfortunately, Y6‐based devices have been reported to be morphologically less stable than ITIC‐based devices, due to the lower thermal transition of Y6 and its variants.^[^ ^16^ ^]^ The metastable blend morphology of Y6‐based devices as well as the complex synthesis of Y‐series acceptors pose challenges for achieving long‐term stable and low‐cost OSC devices.^[^ ^17^ ^]^ To overcome this possible limitation, continued efforts on ITIC‐series (“non‐Y” acceptors) acceptors can help to promote efficiency and enhance stability.^[^ ^18^ ^]^ In this contribution, following our above mentioned C8‐ITIC work, we introduce outer linear octyl chains as a large steric hindrance between the conjugated core and the end groups in an ITIC derivative, and design a SMA {(2Z)‐2‐[(8‐{(E)‐[6,7‐difluoro‐1‐(dicyanomethylidene)‐3‐oxo‐1,3‐dihydro‐2H‐inden‐2‐ylidene]methyl}‐6,6,12,12‐tetraoctyl‐6,12‐dihydro‐4‐octyl‐thieno[3,2‐*b*]thieno[2″,3″:4′,5′]thieno[2′,3′:5,6]‐s‐indaceno[2,1‐*d*]thiophen‐3‐octyl‐2‐yl)methylidene]‐6,7‐difluoro‐3‐oxo‐2,3‐dihydro‐1H‐inden‐1‐ylidene}propanedinitrile (SC8‐IT4F, **Scheme** **1**, bottom left), which has a red‐shifted absorption (λ_max_^film^ = 794 nm) and a similar LUMO level (1.4 eV).^[^ ^19^ ^]^ The single crystal structure of SC8‐IT4F shows high planarity and strong 𝜋–𝜋 stacking. After blending with a WBG polymer poly[(2,6‐(4,8‐bis(5‐(2‐ethylhexyl‐3‐fluoro)thiophen‐2‐yl)‐benzo[1,2‐b:4,5‐*b*′]dithiophene))‐alt‐(5,5‐(1′,3′‐di‐2‐thienyl‐5′,7′‐bis(2‐ethylhexyl)benzo[1′,2′‐*c*:4′,5′‐*c*′]dithiophene‐4,8‐dione)] (PM6), SC8‐IT4F based OSC devices give an outstanding PCE of 15.13%, which is among the best values of OSCs without using Y‐series acceptors. We further analyzed the origin of the difference in photovoltaic performance of SC8‐IT4F and C8‐IT4F via their crystalline properties and blend films morphology. We also demonstrate that these IDTT‐derivatives have better stability than the corresponding L8‐BO‐based devices. This work offers a new avenue for balancing the performance and longevity of organic solar cells with the help of enhanced steric hindrance induced by outer side chains.

## 2 Results and Discussion

The molecular structures of C8‐IT4F and SC8‐IT4F are shown in Scheme 1. C8‐IT4F was synthesized according to the literature method^[^ ^12^ ^]^ and SC8‐IT4F was synthesized according to the route shown. The commercially available precursor **1** was deprotonated with lithium diisopropylamide (LDA) in the free beta position at low temperature. Warming to room temperature resulted in a halogen‐dance rearrangement to afford the more stable isomer lithiated in the alpha position, which was quenched with water to afford isomer **2**. Compound **3** was then obtained via a Friedel‐Crafts acylation with nonanoyl chloride and aluminum chloride. Subsequent treatment with ethyl thioglycolate under basic conditions afforded IDTT‐dicarboxylate with outer octyl side chains **4**. The ester groups on **4** were readily converted to aldehyde groups via a two‐step reduction/oxidation route using lithium aluminum hydride (LiAlH_4_), followed by the oxidation with Dess–Martin periodinane to afford compound **6**. The final acceptor SC8‐IT4F was prepared by the Knoevenagel condensation reaction of **6** and 2‐(5,6‐difluoro‐3‐oxo‐2,3‐dihydro‐1H‐inden‐1‐ylidene)malononitrile (IC‐2F). The chemical structures of C8‐IT4F and SC8‐IT4F were verified by MALDI‐TOF mass spectrometry and proton nuclear magnetic resonance (NMR) spectroscopy (Supporting Information).

**Figure 1:**
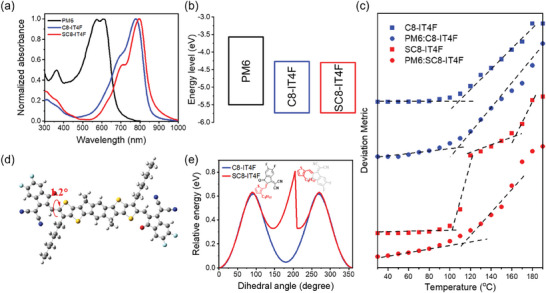
a) UV–vis absorption spectra in film and b) energy level diagrams of PM6, C8‐IT4F, and SC8‐IT4F. c) UV–vis deviation metric (DMT) results of C8‐IT4F and SC8‐IT4F and their blend films. d) Optimal conformation simulated by DFT calculations for SC8‐IT4F in simplified mode and in vacuum. e) Potential energy surface scans of C8‐IT4F and SC8‐IT4F.

UV–vis absorption and photon electron spectroscopy in air (PESA) measurements were performed to correlate the optoelectronic properties with their molecular structures. Compared to the light absorption profile of C8‐IT4F (**Figure** **1**a), the maximum absorption peak (*λ*_max_^film^) of SC8‐IT4F neat film is slightly red‐shifted to 794 nm, which we believe stems from the more rigid structure and better co‐planarity of the end groups as a result of the outer side chains. Furthermore, compared to C8‐IT4F, both solution and thin film absorptions for SC8‐IT4F exhibit distinct shoulders (Figure S13, Supporting Information). The HOMO levels of both acceptors were estimated from the ionization potential measured with PESA, and the LUMO levels were estimated by the addition of the optical band gap (Figure 1b and **Table** **1**, and Figure S14, Supporting Information). Water contact angle measurements on films of both acceptors (Figure S15, Supporting Information) show that both are hydrophobic materials, but only small differences exist (93.5° for C8‐IT4F and 98° for SC8‐IT4F). The results suggest that the structural modification of SC8‐IT4F has a minor influence on the photo‐electronic properties and that both materials can be applied as SMAs to match with high‐performance donor PM6.

**Table 1:** Optical and electrochemical properties of C8‐IT4F and SC8‐IT4F.

Material	*λ*_max_^sol.^ [nm]^a)^	*λ*_max_^film^ [nm]^b)^	*λ*_sh_^sol.→film^ [nm]	*E*_g_^opt^ [eV]^c)^	*E*_LUMO_ [eV]^d)^	*E*_HOMO_ [eV]^d)^
PM6	549	608	59	1.84	−3.57	−5.50
C8‐IT4F	698	778	80	1.46	−4.27	−5.73
SC8‐IT4F	709	794	85	1.43	−4.30	−5.73

^a)^ In chloroform solution (10^−5^ mol L^−1^);

^b)^ Spun‐cast thin films;

^c)^ Estimated from the absorption edges (*E*_g_^opt^ = 1240/*λ*_edge_^film^);

^d)^ HOMO measured by PESA of spun‐cast films (error ± 0.05 eV), and LUMO estimated by the addition of the optical band gap to the HOMO.

The glass transition temperature (*T*_g_) of the neat acceptors and their blends were measured to predict their morphological stability and mechanical properties. Differential scanning calorimetry (DSC) is the most common technique to measure *T*_g_ but tends to work poorly with non‐fullerene acceptors without clear signatures, possibly due to the rigid backbone. Hence we employ a technique based on the UV–vis deviation metric (DMT) results to estimate the *T*_g_ values of these thin films.^[^ ^16^, ^20^ ^]^ Figure 1c shows the analysis for C8‐IT4F, SC8‐IT4F, PM6:C8‐IT4F, and PM6:SC8‐IT4F, with the *T*_g_ estimated to be ca. 100 – 110 °C from the intersection of the two dashed lines in all cases. Typically increasing the alkyl chain density of an acceptor might be expected to lower the *T*_g_, but we do not see that for SC8‐IT4F, which we believe relates to the reduced conformational freedom as a result of the bulky outer side chains. Interestingly, the pure SC8‐IT4F film undergoes two stages of film state transitions, at ≈102 and 162 °C.

The optimal molecular geometry and energy levels of C8‐IT4F and SC8‐IT4F were calculated using density functional theory (DFT). The inner octyl chains attached to cyclopentadiene units were replaced with methyl chains for the sake of simplicity while the outer octyl chains were retained in full. As shown in Figure S16 (Supporting Information), the frontier molecular orbitals delocalize over the whole molecular backbone for both C8‐IT4F and SC8‐IT4F. C8‐IT4F possesses a planar molecular backbone while there is a modest twist angle of 1.2° between the end groups (IC‐2F) and adjacent thiophene of the conjugated core in SC8‐IT4F. To further reveal the influence of outer side chains, relaxed potential surface energy scans were performed to illustrate the impact on the rotatable C−C bonds between the central core and the end groups. As shown in Figure 1e, this demonstrates that the relative energy difference for the two conformers of C8‐IT4F was very small (<0.05 eV), with a relatively high barrier (0.6 eV) to their interconversion. In contrast, for SC8‐IT4F the energy difference for the two conformers is large (ca. 0.35 eV), due to steric clashes in one of the conformers with the outer side chain. The full structures of both conformers are shown in Figure S17 (Supporting Information). In addition, the conversion barrier from the high‐energy conformer to the low‐energy conformer is relatively small (ca. 0.2 eV), suggesting that the majority of SC8‐IT4F molecules adopt a single conformation whilst C8‐IT4F is mixed. The adoption of a single conformation would be expected to reduce energetic disorder in the resulting film.

Solid state order plays a vital role in defining the strength and directionality of electronic coupling interactions in organic molecules.^[^ ^21^ ^]^ Although the blend films of the photoactive layers are often complex, the examination of molecular single‐crystal packing can offer complementary insights that assist in the understanding of charge transport properties. However, the single crystal resources for IDTT‐based SMAs are scarce. Considering their fundamental importance, the single crystals of C8‐IT4F and SC8‐IT4F were grown via slow vapor diffusion. Dark red tabular needles were crystallized for the C8‐IT4F sample. The crystallization of SC8‐IT4F was more problematic, as it tended to afford very thin and easily deformed crystals which were difficult to manipulate. These crystals were also clearly twinned and scattered weakly. After many attempts, we finally employed a solvent‐phase interfacial self‐assembly method to form suitable dark brown crystals (see supporting information for complete detail).

**Figure 2:**
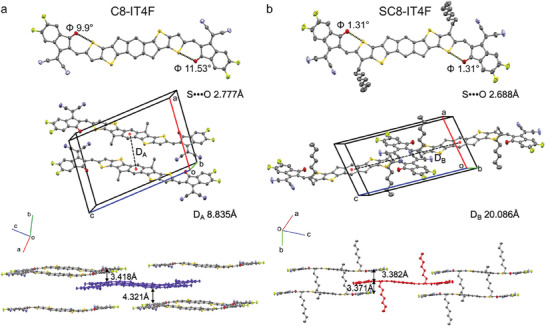
Single crystal structures of a) C8‐IT4F and b) SC8‐IT4F. (Top) π‐face‐on perspective of the single molecules highlighting the intramolecular S•••O distance. (Middle) A unit cell of the indicated SMAs. (Bottom) Crystal packing diagrams illustrate the relationships between an arbitrary central molecule (colored) and near neighbors. Inner alkyl chains on the sp^3^‐hybridized carbon of cyclopentadiene cores and all hydrogen atoms are omitted for ease of viewing.

The crystal structures of C8‐IT4F and SC8‐IT4F are shown in **Figure** **2**. Both SMAs crystallize in triclinic unit cells but differences are observed in their conjugated IDTT core planarity and intermolecular packing. First, the SMAs exhibit intramolecular S•••O═C interactions with smaller distances than the sum of the S and O van der Waals radii (3.25 Å), indicating a conformational lock. While the central IDTT units are overall planar, torsions between the terminal groups and IDTT cores are noted. SC8‐IT4F exhibits a relatively smaller torsion of 1.31° compared to C8‐IT4F (9.9°–11.53°). Both are larger than predicted by the gas‐phase DFT calculations, although the torsion for C8‐IT4F is similar to that previously reported for the single crystal of ITIC and corroborated by simulation of thermal dynamic motions.^[^ ^22^ ^]^ Furthermore, large differences in intermolecular packing are observed within the unit cells. Whilst the asymmetric unit in the structure of C8‐IT4F contains one unique molecule in a general position, for SC8‐IT4F the molecule sits across a center of symmetry and so only half of the molecule is unique. This means that in SC8‐IT4F, both end groups of the molecule will have exactly the same geometry and packing environment, whereas in C8‐IT4F, the flanked end groups are crystallographically distinct and so are not constrained to be identical. For example, in the structure of SC8‐IT4F there is only one unique carbonyl oxygen atom, and so the packing environments of every carbonyl oxygen atom throughout the whole crystal will be the same. In C8‐IT4F by contrast, the carbonyl oxygens atoms at each end of the molecule are crystallographically occupying different environments, indicating geometric asymmetry of the molecule. Likewise, in Figure 2 (Middle), Figures S18 and S19 (Supporting Information), the presence of the inversion center at the middle of the central C_6_ ring in the structure of SC8‐IT4F means that the environment of the “top” face of this C_6_ ring will be identical to the of the “bottom” face, whereas in C8‐IT4F no such symmetry exists. In Figure 2 (Bottom) and Figures S20–S22 (Supporting Information), both C8‐IT4F and SC8‐IT4F present face‐to‐face packings in “brick‐like” planes, separated by their alkyl substituents. In C8‐IT4F, π‐π interactions between the adjacent end groups with interplanar spacings are 3.418 and 4.321 Å. For SC8‐IT4F, the distances are 3.382 and 3.371 Å. Thus, introducing outer side chains on SMAs enhances the stacking of the neighboring molecules in the crystallographic network by promoting π–π end group interactions.

**Figure 3:**
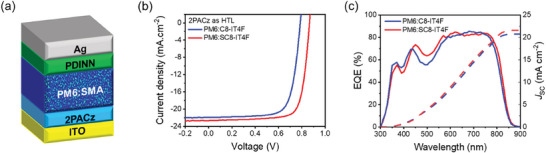
Photovoltaic properties of PM6:SMA OSCs. a) Schematic diagram of the device structure. b) OSC current–voltage (*J–V*) characteristics. c) Corresponding external quantum efficiency (EQE) data for the indicated blends.

**Figure 4:**
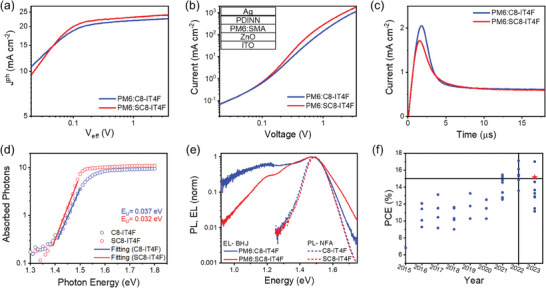
a) Photocurrent density (J_ph_) versus effective bias (V_eff_) characteristics, b) SCLC electron mobility, c) photo‐CELIV, d) PDS, and e) EL of the blend films and PL of neat SMAs excited at 690 nm. f) Scatter plots of PCE in the last 9 years of reported non‐Y SMAs‐based OSCs, including perylene diimide, fused A‐D‐A type, non‐fused SMAs, etc (details in Table S3, Supporting Information).

To investigate the photovoltaic performance of C8‐IT4F and SC8‐IT4F, OSCs were fabricated with the conventional device structure of ITO/(2‐(9H‐carbazol‐9‐yl)ethyl)phosphonic acid (2PACz)/PM6:SMAs/PDINN/Ag, as shown in **Figure** **3**a. PM6 was selected as the electron donor for its good processability, matching energy levels, and complementary absorption with both of the SMAs.^[^ ^23^ ^]^ Various device fabrication conditions, such as photoactive layer thickness and annealing temperatures, were screened to optimize the photovoltaic performance of the OSCs. Figure 3b shows the current density‐voltage (*J–V*) characteristics of the optimized devices, and their photovoltaic parameters are summarized in **Table** **2** for comparison. Notably, the OSCs devices on PM6:SC8‐IT4F exhibit a higher PCE of 15.13% compared to the C8‐IT4F‐based devices, mainly benefiting from the increased *V*_OC_ value. This trend is in agreement with other reported work by introducing outer side chains on Y6.^[^ ^24^ ^]^ We further note that the PCE of 15.13% with SC8‐IT4F as the acceptor in this work is among the highest values for non‐Y SMAs based OSCs. (**Figure** **4**f and Table S3, Supporting Information).

**Table 2:** Photovoltaic performance parameters of PM6:SMA OSCs.

SMA	*V*_OC_ [V]	*J*_SC_ [mA cm^−2^]	Cal. *J*_SC_ [mA cm^−2^]^a)^	FF	PCE [%]^b)^
C8‐IT4F	0.80 (0.78 ± 0.01)	21.92 (21.96 ± 0.17)	20.87	0.74 (0.73 ± 0.01)	12.97 (12.62 ± 0.20)
SC8‐IT4F	0.87 (0.86 ± 0.01)	22.67 (22.59 ± 0.35)	21.51	0.77 (0.76 ± 0.01)	15.13 (14.83 ± 0.22)

^a)^ Calculated from EQE profiles;

^b)^ Average values were obtained from ten independent devices (in brackets).

Examination of the external quantum efficiency (EQE) spectra of the optimal devices (Figure 3c), shows that both C8‐IT4F and SC8‐IT4F‐based devices exhibit strong photo‐response from 350 to 810 nm. The PM6:SC8‐IT4F shows a slightly enhanced EQE ≈500 nm, which might be due to better charge extraction originating from PM6, as a result of the higher blend hole mobility (Figure S23, Supporting Information). In addition, slight variations of the layer thickness, nominally 110 nm for both films, can play a role in the light incoupling. For example, a brief optical simulation of an ITO/PM6:IT4F/PDINO/Ag sample, reveals that even a slight increase of the active layer thickness from 100 to 120 nm increases the absorptance in the 450–500 nm region (Figure S24, Supporting Information). The *J*_SC_ values integrated from the EQE spectra are 20.87 and 21.51 mA cm^−2^ for the OSCs based on C8‐IT4F and SC8‐IT4F, respectively, which agrees well with the measured values obtained from the *J–V* curves.

Both IDTT‐type SMA‐based devices exhibited better thermal and operational stability than PM6:L8‐BO‐based devices (Figure S25, Supporting Information). However, the efficiency of SC8‐IT4F‐based devices unexpectedly degraded faster than that of C8‐IT4F, especially upon thermal annealing at 85 °C. This result is surprising and suggests the apparent two‐phase *T*_g_ transition states of SC8‐IT4F might have a detrimental influence on the morphological stability of PM6:SC8‐IT4F blends.

The exciton dissociation, charge transport, and recombination characteristics in the blend films were investigated to further explore the reason for the different photovoltaic performance of the C8‐IT4F and SC8‐IT4F‐based OSCs. The dependence of photocurrent density (*J*_ph_) on the effective voltage (*V*_eff_) of the devices was investigated, and the results are shown in Figure 4a. PM6:C8‐IT4F and PM6:SC8‐IT4F‐based OSCs show similar charge dissociation probabilities under maximum power point (P_MPP_) (≈84%) and under *J*_SC_ condition (P_C_) (≈96%). However, PM6:SC8‐IT4F‐based devices reach a higher maximum charge generation rate (G_max_) (1.36 × 10^28^ m^−3^ s^−1^) than that of PM6:C8‐IT4F (1.28 × 10^28^ m^−3^ s^−1^). The charge transport properties of the neat and blend films were investigated via the space charge limited current (SCLC) method (Figure 4b and Figures S23 and S26, Supporting Information). The electron mobilities of neat films increased upon moving from C8‐IT4F to SC8‐IT4F, with values of 4.53 × 10^−4^ to 5.69 × 10^−4^ cm^2^ V^−1^ s^−1^. For the blend films, PM6:SC8‐IT4F also displays a higher electron mobility (3.35 × 10^−4^ cm^2^ V^−1^ s^−1^) and hole mobility (1.72 × 10^−4^ cm^2^ V^−1^ s^−1^) than PM6:C8‐IT4F (2.5 × 10^−4^ cm^2^ V^−1^ s^−1^ and 1.36 × 10^−4^ cm^2^ V^−1^ s^−1^ respectively).

The enhanced charge transport is further confirmed via photo‐induced charge‐carrier extraction from linearly increasing voltage (photo‐CELIV) measurements (Figure 4c). The mobility of the charge carriers in OPVs with PM6:SC8‐IT4F is 1.31 × 10^−4^ cm^2^ V^−1^ s^−1^, which is higher than that in PM6:C8‐IT4F cells (1.05 × 10^−4^ cm^2^ V^−1^ s^−1^). The higher electron mobility of the SC8‐IT4F cells is in agreement with the higher structural order and reduced disorder arising from the preference for a single conformer. Further support for the reduced disorder comes from the determination of the Urbach energy using sensitive photothermal deflection spectroscopy (PDS) measurements.^[^ ^25^ ^]^ As shown in (Figure 4d) films of SC8‐IT4F exhibit a smaller Urbach energy than C8‐IT4F films.

The excellent performance of PM6:SC8‐IT4F‐based solar cells, largely comes from the fact, that despite having a slightly smaller optical bandgap (hence maximizing *J*_SC_), SC8‐IT4F enables a larger *V*_OC_ when associated with PM6 in the OSC. We further characterized this combination by probing the energetics of the PM6:SC8‐IT4F and PM6:C8‐IT4F interfaces with electroluminescence (EL), and calculating the additional energy losses leading to *V*_OC_. In EL, we inject electrons in the acceptor and holes in the donor. Their recombination at the D:A interface emits photons whose energy informs us about the energetics of these interface states. As seen in Figure 4e, the PM6:C8‐IT4F interface emits photons of lower energy than the PM6:SC8‐IT4F, indicative of lower energy interface states in the former, which is more strongly constraining *V*_OC_. Comparison with the photoluminescence spectra of pristine films (Figure S28, Supporting Information) shows that the highest energy peak (≈1.5 eV) is actually emitted from the acceptors – either by hybridization of the interface charge transfer state with the acceptor's exciton, or by hole back transfer from the interface to the acceptor.^[^ ^26^ ^]^ In contrast, the lower energy features (0.9–1.3 eV) are most likely due to interface charge transfer state emission. We, however, cannot quantify the fraction of emission originating for the interface, as experience shows that in the related acceptor phenyl(alkyl)ITIC‐4F (PhIT‐4F) the pristine contribution to EL extends further in low energies than its PL.^[^ ^27^ ^]^ For reference, our previous measurements on PM6:PhIT‐4F showed an even larger contribution of CT states to EL, in line with the much larger energy losses observed between PhIT‐4F excitons and the *V*_OC_ of PM6:PhIT‐4F‐based solar cells.^[^ ^27^ ^]^ From those interface states energy, *V*_OC_ is further reduced by recombination losses both radiative and non‐radiative.^[^ ^28^ ^]^ The differences in the EL in the 1.5–1.7 eV region likely relate to small differences in film thickness which can affect light outcoupling. The out‐coupling efficiency is modeled in a representative system in Figure S29 (Supporting Information). It is notable that outcoupling in this region is much more sensitive to thickness changes than in the low energy region ≈1 eV, suggesting thickness differences cannot explain the very difference of emission observed in that region.

The open circuit voltage that would be obtained with radiative losses only (*V_OC,rad_*), for each device was determined to assess the difference in *V*_OC_ loss between the systems using *V_OC,rad_ = kT/q ln(J_SC_/J_0,rad_* +1).^[^ ^29^ ^]^ Where *J_0,rad_* indicates the radiative saturation current density in thermal equilibrium at T = 300 K. *J_0,rad_* is computed by calculating the solar cell response to the blackbody spectrum (*Φ_BB_*) at 300K. As *Φ_BB_* has most of its intensity in the deeper infrared, the solar cell's wavelength‐dependent response (sensitive EQE spectra in Figure S30, Supporting Information), must be extended in that region. To that aim, it is calculated by applying the reciprocity relation to the EL spectra. The losses governed by the non‐radiative recombination (*∆V_OC,nr_*) are calculated by the difference between the *V_OC,rad_*, and the *V*_OC_ extracted from the *J–V* measurement. Table S1 (Supporting Information) summarizes the calculated *V_OC,rad_*, and *∆V_OC,nr_*. Notably, PM6:C8‐IT4F exhibits a non‐radiative *V*_OC_ that is 65 mV higher than PM6:SC8‐IT4F, potentially contributing to a decrease in the *V*_OC_, and therefore, the PCE. Considering that non‐radiative recombination losses are expected to increase for lower optical bandgaps following the energy gap law, this result seems to be counterintuitive.^[^ ^30^ ^]^ It is rationalized however by the fact that recombination proceeds through the interface states, which as we have seen, have a higher energy in PM6:SC8‐IT4F.

**Figure 5:**
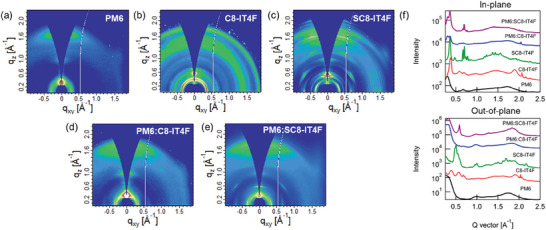
2D GIWAXS images of neat a) PM6, b) C8‐IT4F, c) SC8‐IT4F, d) PM6:C8‐IT4F, e) PM6:SC8‐IT4F blend films and their corresponding f) in‐plane and out‐of‐plane intensity profiles.

We further investigate the molecular orientation and intermolecular packing of PM6, C8‐IT4F and SC8‐IT4F neat films and the two blends using grazing incidence wide‐angle X‐ray scattering (GIWAXS). The 2D GIWAXS patterns are shown in **Figure** **5**a–e and 1D in‐plane (IP) and out‐of‐plane (OoP) profiles are shown in the SI (Figures S31 and S32, Supporting Information). The data from the neat PM6 film (Figure 5a) shows the polymer backbone to be mostly face‐on with an IP lamellar peak at 0.3 Å^−1^ and an OoP π–π stacking peak at 1.74 Å^−1^ (spacing 3.61 Å). The scattering from the neat acceptors C8‐IT4F and SC8‐IT4F are shown in Figure 5b,c, respectively. Both acceptors show multiple peaks indicating the presence of a complex 3D crystalline‐like texture, but they also show notable differences. The rings in the data from C8‐IT4F indicate a 3D powder. In contrast, the arcs in the data from the SC8‐IT4F sample alongside IP lamellar and OoP π–π indicates the presence of partially oriented face‐on 3D crystals. Additionally, most of the reflections in the data from SC8‐IT4F neat sample have double peaks most likely due to reflection of the scattered beam off the substrate or the scattering of the reflected beam. In particular, the π–π stacking peak has some overlap with peaks arising from other reflections as well as a strong background. The strong face‐on orientation of the molecules in the film will result in the highest volume fraction of out‐of‐plane π–π stacks. The π–π stacking peak was therefore identified by taking azimuthal profiles of all peaks in the 1.5–2 Å^−1^ q‐range and choosing the peak with the maximum intensity in the out‐of‐plane direction. Multipeak fitting of the OoP 1‐D profile revealed a π–π stacking peak at 1.90 Å^−1^ (spacing 3.30 Å) for C8‐IT4F sample, and at 1.69 Å^−1^ (spacing 3.72 Å) for the SC8‐IT4F sample.

The blend samples retain all the scattering peaks of the polymer as well as some of the acceptor peaks, but with less definition, indicating a reduced ordering of the acceptor in the blend samples. A notable feature that is present in both neat and blend films of the SC8‐IT4F acceptor is the strong peak near 0.6 Å^−1^ corresponding to a spacing of 10.75 Å in the out‐of‐plane direction that likely originates from vertically stacked face‐on molecules as shown in Figure 2. A peak at a similar q location is also observed in the C8‐IT4F neat data but gets vanishingly small in the C8‐IT4F blend data, which suggests a much smaller volume fraction of such ordered stacks in the C8‐IT4F blend film. The relative degree of crystallinity (rDoC), calculated from the pole figure in the q‐range 0.27–0.4 Å^−1^ covering the lamellar peaks, is significantly higher for the SC8‐IT4F blend compared to the C8‐IT4F blend (ratio ≈ 1:0.47) assuming similar structure factors for the two systems, further confirming the lower degree of ordering in the C8‐IT4F blend. Both blend samples show a prominent π–π stacking peak in the OoP direction suggesting predominantly face‐on orientation of the polymer as well as the SMA in the films. Analysis of the data similar to the neat samples revealed the π–π stacking of the SMA in the PM6:C8‐IT4F and PM6:SC8‐IT4F blends at q locations 1.88 Å (spacing 3.34 Å) and 1.83 Å (spacing 3.43 Å) respectively. The results are summarized in **Table** **3**. The Scherrer coherence length in the SC8‐IT4F neat and blend samples are higher compared to C8‐IT4F samples and correlated with the mobility (Figure 5f) and with FF.^[^ ^31^ ^]^ The IP lamellar peak was also analyzed and similar correlation of the Scherrer coherence length with the electron mobility was found (Figure S32, Supporting Information).

**Table 3:** Spacings and coherence lengths from acceptor π–π stacking peak in GIWAXS data of C8‐IT4F and SC8‐IT4F neat and blend films. Coherence length uncertainty values were calculated from error in FWHM in the fits and given in parenthesis.

Sample	Peak type	q location (Å^−1^)	Spacing (Å)	Coherence length (nm)	Electron mobility (× 10^−4^ cm^2^ V^−1^ s^−1^)
C8‐IT4F neat	π–π	1.90	3.30	5.7 (1.6)	4.53
SC8‐IT4F neat	π–π	1.69	3.72	26.0 (2.9)	5.69
PM6:C8‐IT4F	π–π (SMA)	1.88	3.34	4.8 (0.2)	2.5
PM6:SC8‐IT4F	π–π (SMA)	1.83	3.43	26.1 (0.5)	3.35

These trends were supported by AFM analysis of neat films of C8‐IT4F and SC8‐IT4F as well as their blends with PM6 (Figures S34 and S35, Supporting Information). Both neat films appear highly crystalline but SC8‐IT4F exhibits larger, more fibril domains whereas C8‐IT4F exhibits smaller, more consistent domains. Blending clearly reduces ordering compared to the neat films, with both blends exhibiting a similar surface morphology.

## 3 Conclusion

In summary, a small molecule acceptor SC8‐IT4F was designed and synthesized by introducing outer linear octyl chains on the β position of terminal thiophene units of the conjugated IDTT core. This side chain engineering results in a small reduction in optical band gap compared to the reference molecule C8‐IT4F, beneficial for achieving higher *J*_SC_. Despite the band gap reduction, non‐radiative losses are reduced in SC8‐IT4F blends as a result of higher energy interface states, resulting in higher *V*_OC_ compared to C8‐IT4F. The terminal side chains induce a strong preference for a single conformer, reducing disorder and promoting closer intermolecular packing and higher electron mobility. The higher order was retained in blend films, with a higher average crystallite size observed for the PM6:SC8‐IT4F blend film compared to the PM6:C8‐IT4F blend. Consequently, the blend films based on PM6:SC8‐IT4F display high charge carrier mobility, longer carrier lifetime, and reduced recombination. A champion PCE of 15.13% with a larger *V*_OC_ of 0.87 V was realized for PM6:SC8‐IT4F‐based devices, demonstrating this outer side chain modulation is an effective approach to improve the performance of IDTT‐analog SMAs.

## Conflict of Interest

The authors declare no conflict of interest.

## Author Contributions

M.H. and Q.H. conceptualized the study. Q.H. synthesized the C8‐IT4F and SC8‐IT4F, performed UV–vis and UV–vis deviation metric analysis, and prepared single crystals. W.T.H. prepared the photovoltaic devices and performed *J–V*, mobility, and stability experiments. X.H. and B.D. performed the DFT calculations. S.M., B.L., and H.A. performed the GIWAXS experiments and analyzed the data. M.A. performed the EL measurement and W.A. performed the PDS measurement. J.G. guided the analysis and interpretation of the data for those two measurements. C.S.P.D. carried out the PL measurement and its analysis. M.A., W.A., J.G., and C.S.P.D. were supervised by F.L., J.L., and P.K. performed the PESA, AFM and contact angle measurements and analyzed the data. Z.F. synthesized the C8‐IDTT‐CHO. A.J.P.W. performed the single‐crystal experiments. The manuscript was written by Q.H. with input from the other authors.

## Supporting information



Supporting Information

## Data Availability

The data that support the findings of this study are available from the corresponding author upon reasonable request.

## References

[1] a) G. Zhang , F. R. Lin , F. Qi , T. Heumuller , A. Distler , H. J. Egelhaaf , N. Li , P. C. Y. Chow , C. J. Brabec , A. K. Jen , H. L. Yip , Chem. Rev. 2022, 122, 14180; 35929847 10.1021/acs.chemrev.1c00955

[2] a) J. Wang , P. Xue , Y. Jiang , Y. Huo , X. Zhan , Nat. Rev. Chem. 2022, 6, 614; 37117709 10.1038/s41570-022-00409-2

[3] a) C. Yan , S. Barlow , Z. Wang , H. Yan , A. K. Y. Jen , S. R. Marder , X. Zhan , Nat. Rev. Mater. 2018, 3, 18003;

[4] a) Y. Lin , F. Zhao , Q. He , L. Huo , Y. Wu , T. C. Parker , W. Ma , Y. Sun , C. Wang , D. Zhu , A. J. Heeger , S. R. Marder , X. Zhan , J. Am. Chem. Soc. 2016, 138, 4955; 27015115 10.1021/jacs.6b02004

[5] J. Wang , X. Zhan , Acc. Chem. Res. 2021, 54, 132. 33284599 10.1021/acs.accounts.0c00575

[6] J. Wang , X. Zhan , Chin. J. Chem. 2022, 40, 1592.

[7] a) J. Yuan , Y. Zou , Org. Electron. 2022, 102, 106436;

[8] a) H. Lai , F. He , Adv. Energy Mater. 2020, 10, 2002678;

[9] a) M. Zhang , B. Chang , R. Zhang , S. Li , X. Liu , L. Zeng , Q. Chen , L. Wang , L. Yang , H. Wang , J. Liu , F. Gao , Z. G. Zhang , Adv. Mater. 2023, 36, 2308606; 10.1002/adma.202308606 37816121

[10] a) Q. He , M. Shahid , X. Jiao , E. Gann , F. D. Eisner , T. Wu , Z. Fei , T. D. Anthopoulos , C. R. McNeill , M. Heeney , ACS Appl. Mater. Interfaces 2020, 12, 9555; 31999092 10.1021/acsami.0c00981

[11] a) P. Wang , F. Bi , Y. Li , C. Han , N. Zheng , S. Zhang , J. Wang , Y. Wu , X. Bao , Adv. Funct. Mater. 2022, 32, 2200166;

[12] Z. Fei , F. D. Eisner , X. Jiao , M. Azzouzi , J. A. Rohr , Y. Han , M. Shahid , A. S. R. Chesman , C. D. Easton , C. R. McNeill , T. D. Anthopoulos , J. Nelson , M. Heeney , Adv. Mater. 2018, 30, 1705209. 10.1002/adma.201705209 29315933

[13] a) M. Deng , X. Xu , Y. Duan , L. Yu , R. Li , Q. Peng , Adv. Mater. 2023, 35, 2210760; 10.1002/adma.202210760 36599710

[14] C. Li , J. Zhou , J. Song , J. Xu , H. Zhang , X. Zhang , J. Guo , L. Zhu , D. Wei , G. Han , J. Min , Y. Zhang , Z. Xie , Y. Yi , H. Yan , F. Gao , F. Liu , Y. Sun , Nat. Energy 2021, 6, 605.

[15] a) Y. Li , T. Li , Y. Lin , Mater. Chem. Front. 2021, 5, 2907;

[16] a) M. Ghasemi , N. Balar , Z. Peng , H. Hu , Y. Qin , T. Kim , J. J. Rech , M. Bidwell , W. Mask , I. McCulloch , W. You , A. Amassian , C. Risko , B. T. O'Connor , H. Ade , Nat. Mater. 2021, 20, 525; 33432145 10.1038/s41563-020-00872-6

[17] Y. Firdaus , Q. He , L. Muliani , E. S. Rosa , M. Heeney , T. D. Anthopoulos , Adv. Nat. Sci.: Nanosci. Nanotechnol. 2022, 13, 025001.

[18] a) R. Zheng , C. Zhang , A. Zhang , J. Xue , X. Xu , Y. Liu , C. J. Su , W. Ma , C. Yang , Z. Bo , ACS Appl. Mater. Interfaces 2023, 15, 4275; 36645327 10.1021/acsami.2c22292

[19] Z. Zhang , J. Yu , X. Yin , Z. Hu , Y. Jiang , J. Sun , J. Zhou , F. Zhang , T. P. Russell , F. Liu , W. Tang , Adv. Funct. Mater. 2018, 28, 1705095.

[20] S. E. Root , M. A. Alkhadra , D. Rodriquez , A. D. Printz , D. J. Lipomi , Chem. Mater. 2017, 29, 2646.

[21] T. J. Aldrich , M. Matta , W. Zhu , S. M. Swick , C. L. Stern , G. C. Schatz , A. Facchetti , F. S. Melkonyan , T. J. Marks , J. Am. Chem. Soc. 2019, 141, 3274. 30672702 10.1021/jacs.8b13653

[22] G. Han , Y. Guo , X. Song , Y. Wang , Y. Yi , J. Mater. Chem. C 2017, 5, 4852.

[23] M. Zhang , X. Guo , W. Ma , H. Ade , J. Hou , Adv. Mater. 2015, 27, 4655. 26173152 10.1002/adma.201502110

[24] Z. Luo , Y. Gao , H. Lai , Y. Li , Z. Wu , Z. Chen , R. Sun , J. Ren , C. e. Zhang , F. He , H. Y. Woo , J. Min , C. Yang , Energy Environ. Sci. 2022, 15, 4601.

[25] A. J. Kronemeijer , V. Pecunia , D. Venkateshvaran , M. Nikolka , A. Sadhanala , J. Moriarty , M. Szumilo , H. Sirringhaus , Adv. Mater. 2014, 26, 728. 24170627 10.1002/adma.201303060 PMC4230477

[26] D. Qian , Z. Zheng , H. Yao , W. Tress , T. R. Hopper , S. Chen , S. Li , J. Liu , S. Chen , J. Zhang , X.‐K. Liu , B. Gao , L. Ouyang , Y. Jin , G. Pozina , I. A. Buyanova , W. M. Chen , O. Inganäs , V. Coropceanu , J.‐L. Bredas , H. Yan , J. Hou , F. Zhang , A. A. Bakulin , F. Gao , Nat. Mater. 2018, 17, 703. 30013057 10.1038/s41563-018-0128-z

[27] S. Karuthedath , J. Gorenflot , Y. Firdaus , N. Chaturvedi , C. S. P. De Castro , G. T. Harrison , J. I. Khan , A. Markina , A. H. Balawi , T. A. D. Peña , W. Liu , R.‐Z. Liang , A. Sharma , S. H. K. Paleti , W. Zhang , Y. Lin , E. Alarousu , S. Lopatin , D. H. Anjum , P. M. Beaujuge , S. De Wolf , I. McCulloch , T. D. Anthopoulos , D. Baran , D. Andrienko , F. Laquai , Nat. Mater. 2021, 20, 378. 33106652 10.1038/s41563-020-00835-x

[28] K. Vandewal , K. Tvingstedt , A. Gadisa , O. Inganäs , J. V. Manca , Phys. Rev. B. 2010, 81, 125204.

[29] T. H. Lee , S. Y. Park , X. Du , S. Park , K. Zhang , N. Li , S. Cho , C. J. Brabec , J. Y. Kim , ACS Appl. Mater. Interfaces 2020, 12, 55945. 33270428 10.1021/acsami.0c16854

[30] J. Benduhn , K. Tvingstedt , F. Piersimoni , S. Ullbrich , Y. Fan , M. Tropiano , K. A. McGarry , O. Zeika , M. K. Riede , C. J. Douglas , S. Barlow , S. R. Marder , D. Neher , D. Spoltore , K. Vandewal , Nat. Energy 2017, 2, 17053.

[31] H. Hu , K. Jiang , P. C. Y. Chow , L. Ye , G. Zhang , Z. Li , J. H. Carpenter , H. Ade , H. Yan , Adv. Energy Mater. 2018, 8, 1701674.

[32] H. Alexander , B. Wim , G. James , S. Eric , G. Eliot , K. Rick , M. Alastair , C. Matthew , R. Bruce , P. Howard , J. Phys.: Conf. Ser. 2010, 247, 012007.

