# Supplementary material for: Significant Efficiency Enhancements in Non‐Y Series Acceptors by the Addition of Outer Side Chains
Source: Adv Sci (Weinh). 2025 Jan 22;12(11):2414042. doi: 10.1002/advs.202414042 (PMC11923974; doi:10.1002/advs.202414042)
Supplement: Supplementary file 1 — Supporting Information [file ADVS-12-2414042-s001.docx]

Supporting Information

Significant efficiency enhancements in non-Y series acceptors by the addition of outer side chains

Qiao He, Wisnu Tantyo Hadmojo, Xiantao Hu, Subhrangsu Mukherjee, Maryam Alqurashi, Wejdan Althobaiti, Catherine S. P. De Castro, Byongkyu Lee, Bowen Ding, Joel Luke, Panagiota Kafourou, Zhuping Fei, Andrew J. P. White, Julien Gorenflot, Florian Glöcklhofer, Frédéric Laquai, Harald Ade, Thomas D. Anthopoulos, and Martin Heeney*

**Synthesis**

All reactions were carried out in oven-dried glassware under nitrogen at room temperature using solvents and reagents as commercially supplied, unless otherwise stated. C8-IDTT-CHO was synthesized according to a reported literature.^[1]^ Petroleum ether refers to the fraction boiling at 40-60 °C. All the NMR spectra were recorded on a Bruker AV-400 (400 MHz), using the residual solvent resonance of CDCl_3_ and are given in ppm.

**3,8-dibromo-4,4,9,9-tetraoctyl-4,9-dihydro-s-indaceno[1,2-b:5,6-b']dithiophene (2)**

To a solution of 2,7-dibromo-4,4,9,9-tetraoctyl-4,9-dihydro-s-indaceno[1,2-b:5,6-b']dithiophene 1 (720 mg, 0.82 mmol) in anhydrous THF (80 mL) at −78 °C was added a solution of lithium diisopropylamide (LDA) (3.3 mL of a 1 M solution in THF, 3.28 mmol) dropwise. The mixture was stirred for 1h at this temperature, and then warmed to RT for overnight. Water (10 mL) was added dropwise and stirred for 30 min. Then the mixture was extracted with DCM and water three times. The combined organics were dried by MgSO_4_, filtered and concentrated under reduced pressure. The residue was purified by silica gel chromatography (eluent: petroleum ether) to afford a yellow crystal (578 mg, yield: 80%). ^1^H NMR (400 MHz, CDCl_3_, δ): 7.23 (s, 2H), 7.15 (s, 2H), 2.38-1.88 (m, 8H), 1.22-1.09 (m, 48H), 0.92-0.51 (m, 12H). MALDI-TOF: 873.0 (M^+^).

Figure S1. ^1^H NMR spectrum of Compound **2** in CDCl_3_.


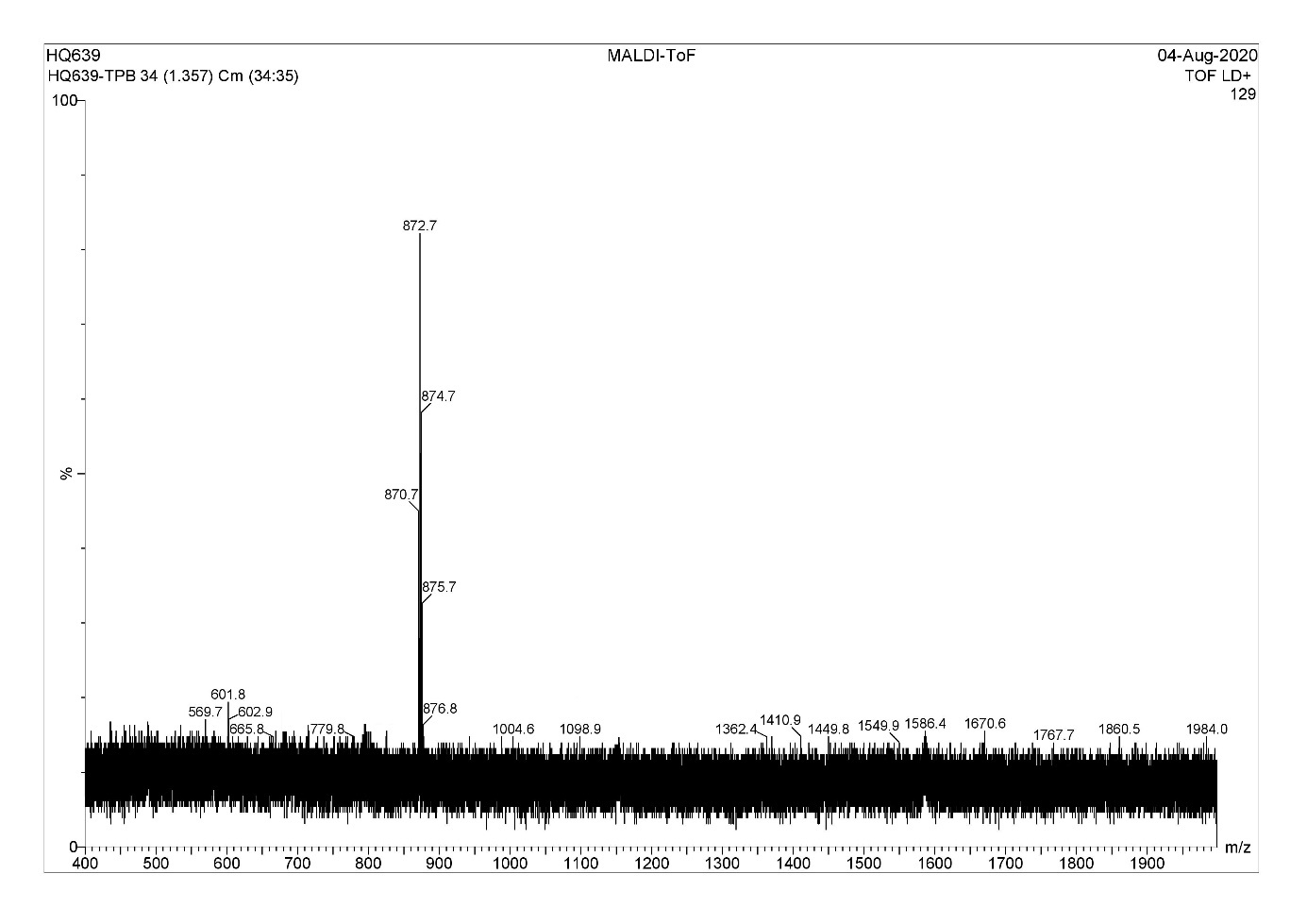


Figure S2. MALDI-TOF MS plot of Compound **2**.

**1,1'-(3,8-dibromo-4,4,9,9-tetraoctyl-4,9-dihydro-s-indaceno[1,2-b:5,6-b']dithiophene-2,7-diyl)bis(nonan-1-one) (3)**

To a solution of compound 2 (570 mg, 0.65 mmol) and nonanoyl chloride (254 mg, 1.44 mmol) in anhydrous DCM was added aluminum chloride (192 mg, 1.44 mmol) in one portion. The mixture was stirred for 6h and water (20 mL) was slowly added to quench the reaction. The mixture was extracted with DCM and the combined organics were dried by MgSO_4_, filtered and concentrated under reduced pressure. The residue was purified by silica gel chromatography (eluent: DCM:petroleum ether = 1:2) to afford a yellow orange solid (512 mg, yield: 68%). ^1^H NMR (400 MHz, CDCl_3_, δ): 7.38 (s, 2H), 3.12-2.96 (m, 4H), 2.56-2.36 (m, 4H), 2.02-1.68 (m, 8H), 1.48-0.94 (m, 68H), 0.94-0.41 (m, 18H). MALDI-TOF: 1153.1 (M^+^).

Figure S3. ^1^H NMR spectrum of Compound **3** in CDCl_3_.

**diethyl(6,6,12,12-tetraoctyl-6,12-dihydro-thieno[3,2-b]thieno[2'',3'':4',5']thieno[2',3':5,6]-s-indaceno[2,1-d]thiophene-3,9-dioctyl-2,8-dicarboxylate) (4)**

Ethyl mercaptoacetate (0.3 mL, 2.6 mmol) was added dropwise to a mixture of compound 3 (510 mg) and K_2_CO_3_ (360 mg, 2.6 mmol) in anhydrous DMF (50 mL) at 65 °C. After addition, the mixture was stirred at this temperature for 24 h, and then poured into water (100 mL) and extracted with DCM (3 × 100 mL). The combined organics were dried by MgSO_4_, filtered and concentrated under reduced pressure. The residue was purified by silica gel chromatography (eluent: DCM:petroleum ether = 1:2) to afford a yellow orange oil (405 mg, yield: 76%). ^1^H NMR (400 MHz, CDCl_3_, δ): 7.35 (s, 2H), 4.41-4.35 (m, 4H), 3.22-3.18 (m, 4H), 2.22-1.90 (m, 8H), 1.86-1.71 (m, 4H), 1.52-0.98 (m, 68H), 0.96-0.66 (m, 24H). MALDI-TOF: 1195.3 (M^+^).

Figure S4. ^1^H NMR spectrum of Compound **4** in CDCl_3_.


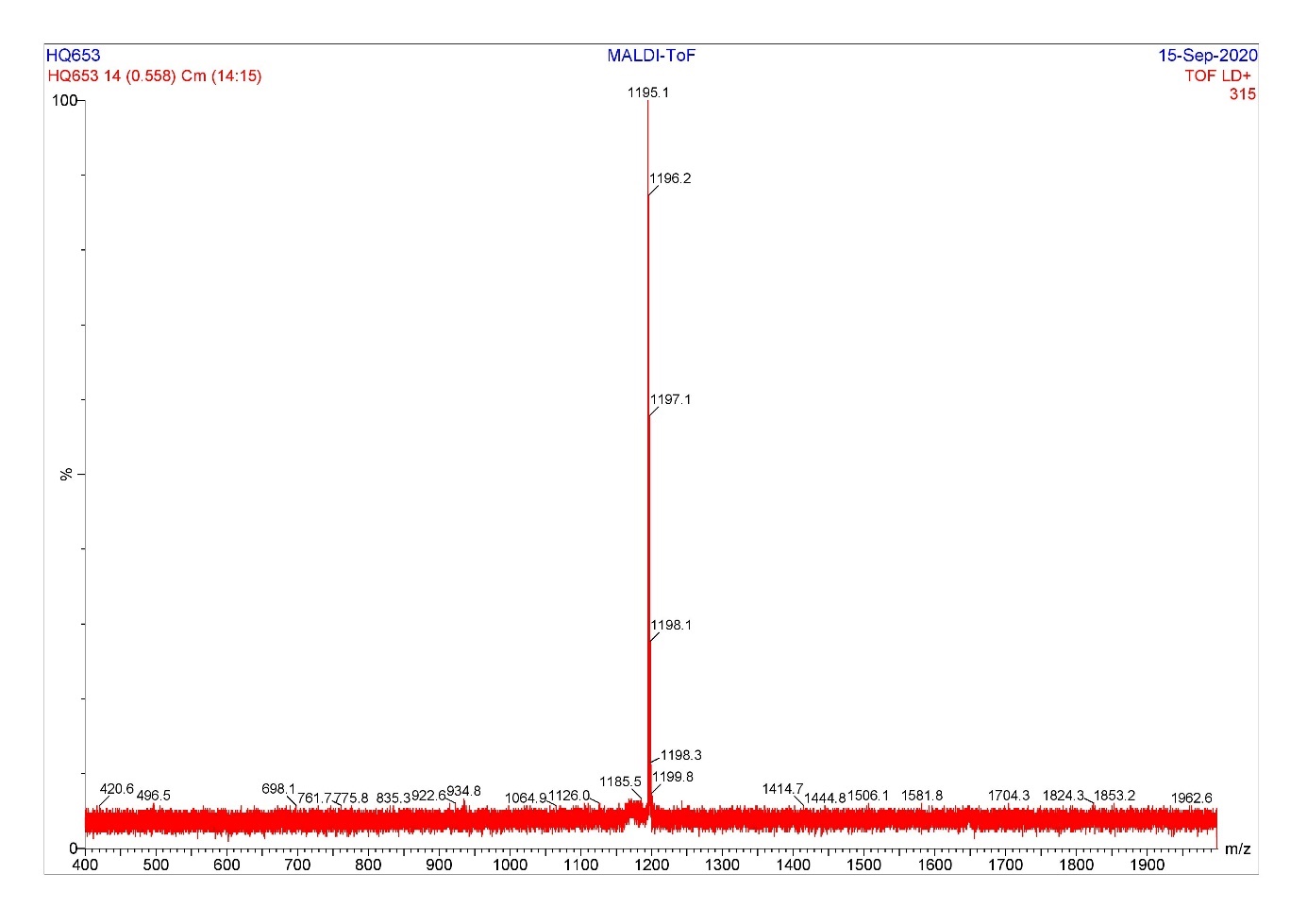


Figure S5. MALDI-TOF MS plot of Compound **4**.

**6,6,12,12-tetraoctyl-6,12-dihydro-thieno[3,2-b]thieno[2'',3'':4',5']thieno[2',3':5,6]-s-indaceno[2,1-d]thiophen-3,9-dioctyl-2,8-diyldimethanol (5)**

A solution of LiAiH_4_ (1.9 mL of a 1M solution in THF, 1.88 mmol) was added dropwise into compound 4 (400 mg, 0.33 mmol) in THF (40 mL). After stirred for 2h, the mixture was cooled to 0 °C, and then saturated NH_4_Cl solution (10 mL) and KOH (3M, 10 mL) were added carefully. The mixture was extracted with DCM (3 × 30 mL). The combined organics were dried by MgSO_4_, filtered and concentrated under reduced pressure. The crude product was used directly without further purification.

**6,6,12,12-tetraoctyl-6,12-dihydro-thieno[3,2-b]thieno[2'',3'':4',5']thieno[2',3':5,6]-s-indaceno[2,1-d]thiophen-3,9-dioctyl-2,8-dicarbaldehyde (6)**

To a solution of compound 5 in DCM (30 mL) at 0 °C, Dess-Martin periodinane (395 mg, 0.93 mmol) was added in one portion. After addition, the cold bath was removed and the mixture was allowed to warm to RT and stirred for 2h. Saturated Na_2_SO_3_ solution (20 mL) was added, and the mixture was extracted with DCM (3 × 30 mL). The combined organics were dried by MgSO_4_, filtered and concentrated under reduced pressure. The residue was purified by silica gel chromatography (eluent: DCM:petroleum ether = 1:1) to afford orange solid (240 mg, yield: 65%). ^1^H NMR (400 MHz, CDCl_3_, δ): 10.13 (s, 2H), 7.44 (s, 2H), 3.25-3.06 (m, 4H), 2.25-1.98 (m, 8H), 1.96-1.82 (m, 4H), 1.52-0.98 (m, 68H), 0.96-0.66 (m, 18H). MALDI-TOF: 1107.1 (M^+^).

Figure S6. ^1^H NMR spectrum of Compound **6** in CDCl_3_.

**{(2Z)-2-[(8-{(E)-[6,7-difluoro-1-(dicyanomethylidene)-3-oxo-1,3-dihydro-2H-inden-2-ylidene]methyl-6,6,12,12-tetraoctyl-6,12-dihydro-thieno[3,2-b]thieno[2'',3'':4',5']thieno[2',3':5,6]-s-indaceno[2,1-d]thiophen-3,9-dioctyl-2-yl)methylidene]-6,7-difluoro-3-oxo-2,3-dihydro-1H-inden-1-ylidene}propanedinitrile (SC8-IT4F)**

To a mixture of 2-(5,6-difluoro-3-oxo-2,3-dihydro-1H-inden-1-ylidene)malononitrile (IC-2F) (96.6 mg, 0.42 mmol) and compound 6 (155 mg, 0.14 mmol) in CHCl_3_ (20 mL) was added pyridine (0.5 mL). After addition, the mixture was heated to 65 °C and stirred overnight, and then poured into water (30 mL) and extracted with DCM (3 × 30 mL). The combined organics were dried by MgSO_4_, filtered and concentrated under reduced pressure. The residue was purified by silica gel chromatography (eluent: DCM:petroleum ether = 1:1) to afford a dark blue solid (167 mg, yield: 78%). ^1^H NMR (400 MHz, CDCl_3_, δ): 9.13 (s, 2H), 8.59-8.55 (m, 2H), 7.73-7.69 (m, 2H), 7.49 (s, 2H), 3.22-3.18 (m, 4H), 2.34-2.02 (m, 8H), 1.92-1.78 (m, 4H), 1.42-0.96 (m, 68H), 0.95-0.65 (m, 18H). ^19^F NMR (400 MHz, CDCl_3_, δ): -123.11 (d, J = 20 Hz), -124.29 (d, J = 20 Hz). MALDI-TOF: 1532.0 (M^+^).

Figure S7. ^1^H NMR spectrum of Compound **SC8-IT4F** in CDCl_3_.

Figure S8. ^19^F NMR spectrum of Compound **SC8-IT4F** in CDCl_3_.


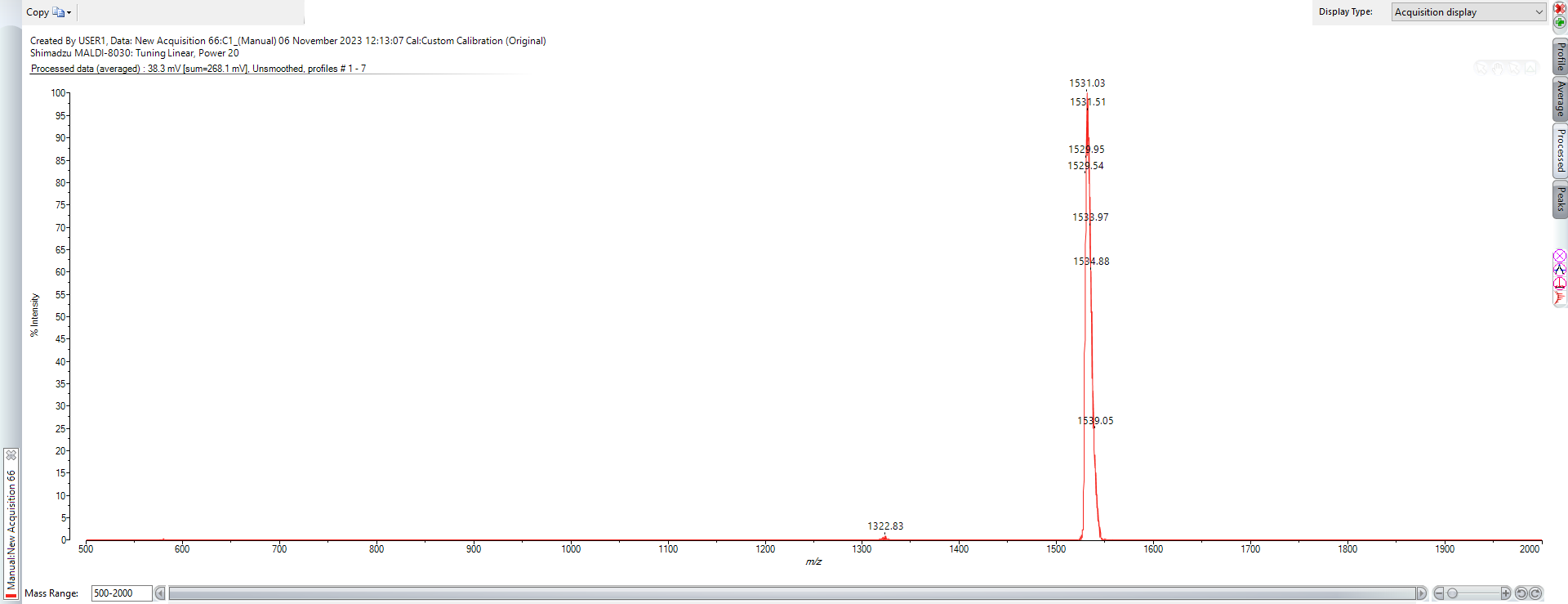


Figure S9. MALDI-TOF MS plot of Compound **SC8-IT4F**.

**{(2Z)-2-[(8-{(E)-[6,7-difluoro-1-(dicyanomethylidene)-3-oxo-1,3-dihydro-2H-inden-2-ylidene]methyl}-6,6,12,12-tetraoctyl-6,12-dihydro-thieno[3,2-b]thieno[2'',3'':4',5']thieno[2',3':5,6]-s-indaceno[2,1-d]thiophen-2-yl)methylidene]-6,7-difluoro-3-oxo-2,3-dihydro-1H-inden-1-ylidene}propanedinitrile (C8-IT4F)**

To a mixture of 2-(5,6-difluoro-3-oxo-2,3-dihydro-1H-inden-1-ylidene)malononitrile (IC-2F) (96.6 mg, 0.42 mmol) and compound C8-IDTT-CHO^[1]^ (155 mg, 0.14 mmol) in CHCl_3_ (20 mL) was added pyridine (0.5 mL). After addition, the mixture was heated to 65 °C and stirred for overnight, and then poured into water (30 mL) and extracted with DCM (3 × 30 mL). The combined organics were dried by MgSO_4_, filtered and concentrated under reduced pressure. The residue was purified by silica gel chromatography (eluent: DCM:petroleum ether = 1:1) to afford a dark blue solid (167 mg, yield: 81%). ^1^H NMR (400 MHz, CDCl_3_, δ): 8.97 (s, 2H), 8.58-8.53 (m, 2H), 8.24 (s, 2H), 7.74-7.70 (m, 2H), 7.51 (s, 2H), 2.31-2.08 (m, 8H), 1.68-0.99 (m, 48H), 0.95-0.65 (m, 12H). ^19^F NMR (400 MHz, CDCl_3_, δ): -122.41 (d, J = 20 Hz), -123.61 (d, J = 20 Hz). MALDI-TOF: 1307.1 (M^+^).

Figure S10. ^1^H NMR spectrum of Compound **C8-IT4F** in CDCl_3_.

Figure S11. ^19^F NMR spectrum of Compound **C8-IT4F** in CDCl_3_.


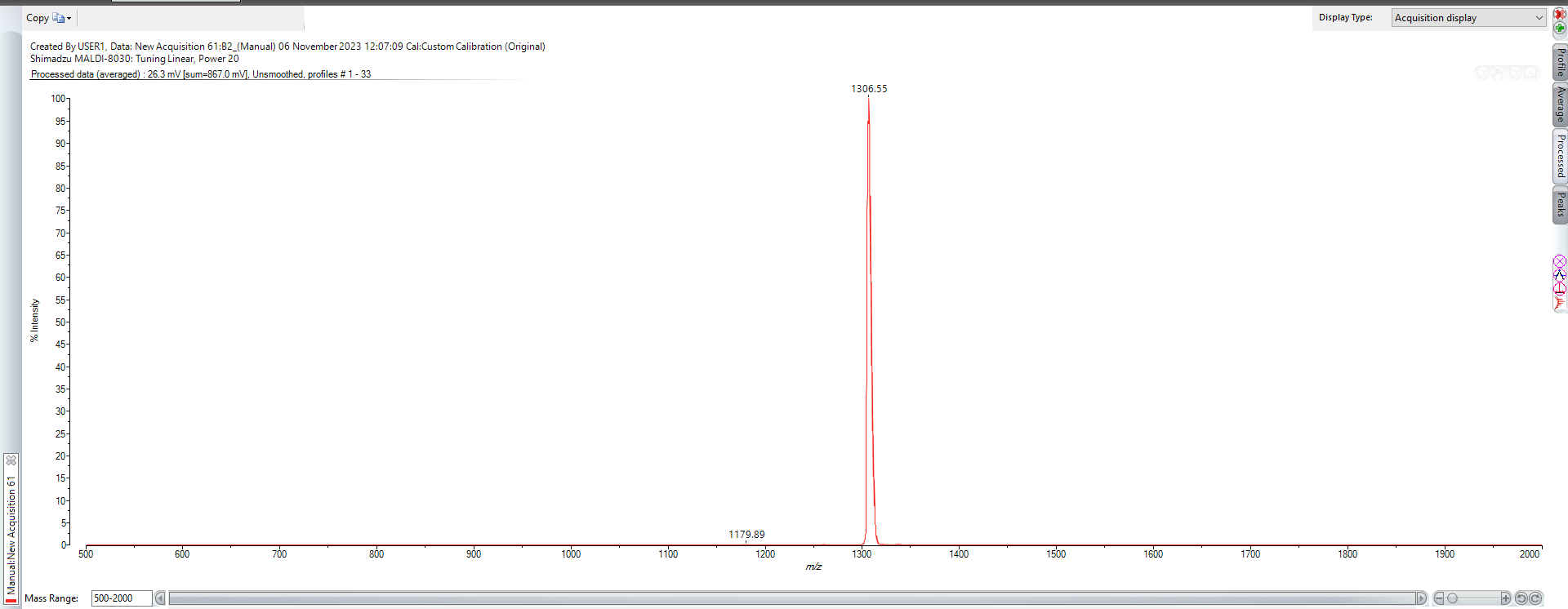


Figure S12. MALDI-TOF MS plot of Compound **C8-IT4F**.

**Characterizations**

UV-vis spectra were recorded on an Agilent Cary 60 UV-vis spectrometer. Photoelectron spectroscopy in air measurements were recorded with a Riken Keiki AC-2 spectrometer on thin films (prepared by spin-coating 10 mg/mL chloroform solutions at 2000 rpm on ITO coated glass substrate). The cube root of the photoemission signal is used in accordance with Fowler’s analysis for semiconductors. Density functional theory (DFT) calculations were modeled using Gaussian at the B3LYP/6-31G* level. The inner side chains on the cyclopentadiene were modified to methyl groups to simplify the calculations.

UV-vis spectroscopy to measure the *T*_g_ of organic thin films

UV-vis spectroscopy is used to measure the changes in the absorption spectrum which are caused by molecular rearrangement when heated past *T*_g_, corresponding to the formation of aggregates. Herein, we measured the absorption spectra of the C8-IT4F, SC8-IT4F and blend thin films with the increasing thermal annealing temperature and for quantitative analysis, we utilize the same method as reported by Root et.al.^[2]^ Deviation metric (DMT) is defined as the sum of the squared deviation in the absorbance between as-cast and annealed films (at annealing temperature, T), where I_RT_(λ) and I_T_ (λ) are the normalized absorption intensities of the as-cast (room temperature) and annealed films, respectively, λ is the wavelength, and λ_min_ and λ_max_ are the lower and upper bounds of the optical sweep, respectively.


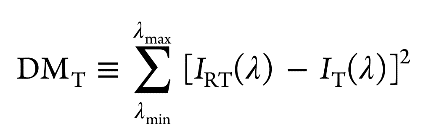
 Equation (S1)

Single-Crystal X-ray Diffraction Analysis

Crystal Growth: C8-IT4F (2 mg) sample was dissolved in CHCl_3_ (1 mL) in a 2 dram vial. This vial was placed into a 10 dram vial containing Methanol (5 mL; 99.8%, Sigma Aldrich). The larger vial was then sealed tightly and left undisturbed at room temperature until crystal growth occurred (2 weeks). The single crystal of SC8-IT4F can be obtained by a solution-processed solvent-phase interfacial self-assembly method. A SC8-IT4F solution in CHCl_3_ (2 mL, 0.5 mg mL^−1^) is prepared firstly in a screw-type 10 dram vial, then 6 mL hexane is added very slowly onto the surface of the CHCl_3_ solution drop by drop using a syringe. After 2 weeks’ standing at room temperature, single crystals can be obtained.

The X-ray crystal structure of C8-IT4F

*Crystal data for* **C8-IT4F**: C_78_H_78_F_4_N_4_O_2_S_4_·2(CHCl_3_), *M* = 1546.42, triclinic, *P*-1 (no. 2), *a* = 13.8495(7), *b* = 14.6046(7), *c* = 19.7736(7) Å, α = 98.206(3), β = 96.884(3), γ = 97.665(4)°, *V* = 3883.7(3) Å^3^, *Z* = 2, *D*_c_ = 1.322 g cm^–3^, μ(Cu-Kα) = 3.494 mm^–1^, *T* = 173 K, dark red tabular needles, Agilent Xcalibur PX Ultra A diffractometer; 14763 independent measured reflections (*R*_int_ = 0.0471), *F*^2^ refinement,^[3]^ *R*_1_(obs) = 0.0624, *wR*_2_(all) = 0.1941, 9490 independent observed absorption-corrected reflections [|*F*_o_| > 4σ(|*F*_o_|), completeness to θ_full_(67.7°) = 98.1%], 960 parameters. CCDC 2271670.

The terminal –C_4_H_9_ portion of the C79-based –C_8_H_17_ chain, and the C90- and C100-based included chloroform solvent molecules in the structure of **C8-IT4F** were all found to be disordered, and in each case two orientations were identified of *ca*. 73:27, 90:10 and 85:15% occupancy respectively. The geometries of each pair of orientations were optimised, the thermal parameters of adjacent atoms were restrained to be similar, and only the non-hydrogen atoms of the major occupancy orientations were refined anisotropically (those of the minor occupancy orientations were refined isotropically).

The X-ray crystal structure of SC8-IT4F

*Crystal data for* **SC8-IT4F**: C_94_H_110_F_4_N_4_O_2_S_4_·1.5(CHCl_3_), *M* = 1711.15, triclinic, *P*-1 (no. 2), *a* = 10.3845(5), *b* = 11.6117(5), *c* = 20.0858(9) Å, α = 88.830(3), β = 76.682(4), γ = 80.502(4)°, *V* = 2324.14(19) Å^3^, *Z* = 1 [*C_i_* symmetry], *D*_c_ = 1.223 g cm^–3^, μ(Cu-Kα) = 2.578 mm^–1^, *T* = 173 K, blacks blocks, Agilent Xcalibur PX Ultra A diffractometer; 8858 independent measured reflections (*R*_int_ = 0.0305), *F*^2^ refinement,^[3]^ *R*_1_(obs) = 0.0445, *wR*_2_(all) = 0.1317, 6824 independent observed absorption-corrected reflections [|*F*_o_| > 4σ(|*F*_o_|), completeness to θ_full_(67.7°) = 98.1%], 527 parameters. CCDC 2271671.

The structure of **SC8-IT4F** was found to sit across a centre of symmetry at the middle of the central C_6_ ring. When refined freely, the occupancy of the C60-based included chloroform solvent molecule settled at *ca*. 76%, so this was fixed at 75% in later refinements for simplicity.


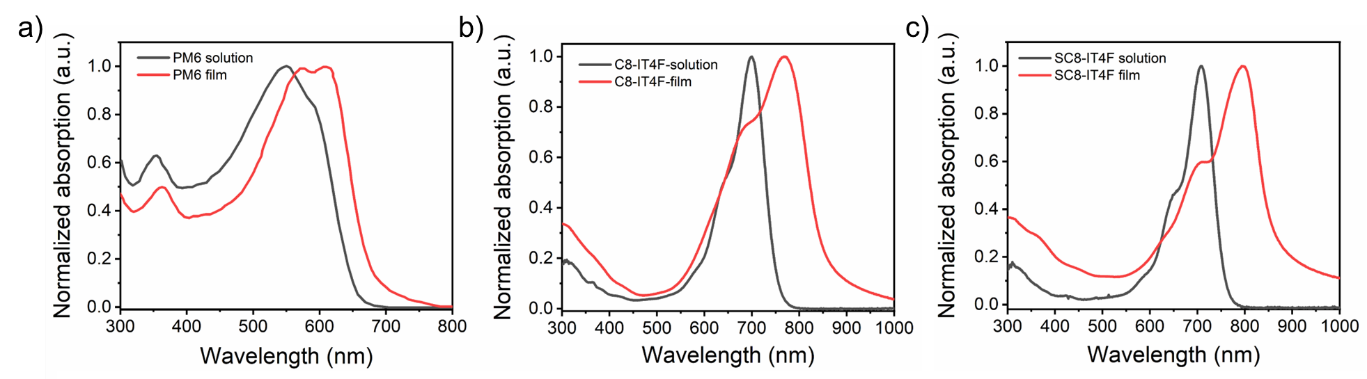


Figure S13. Normalized solution and film UV-vis absorption of PM6, C8-IT4F and SC8-IT4F.

Figure S14. PESA spectra of C8-IT4F and SC8-IT4F films. Identical onset in both cases.


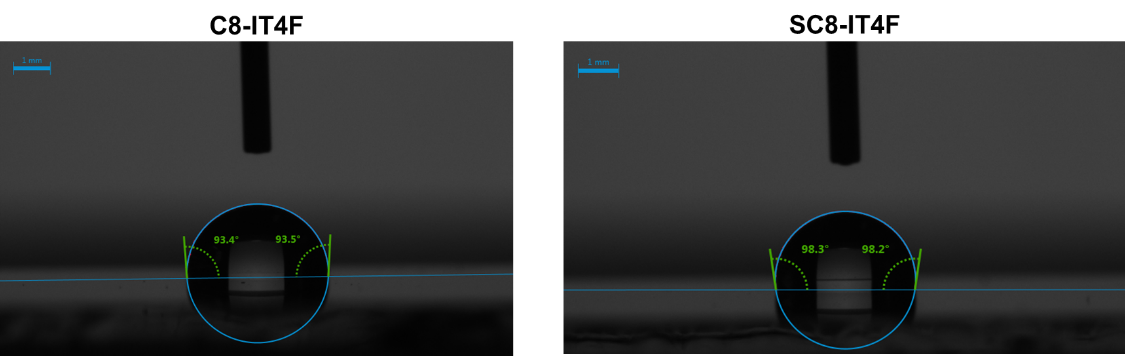


Figure S15. Contact angle images of C8-IT4F and SC8-IT4F films with water on top. The addition of alkyl chains makes films slightly more hydrophobic from contact angle measurements.


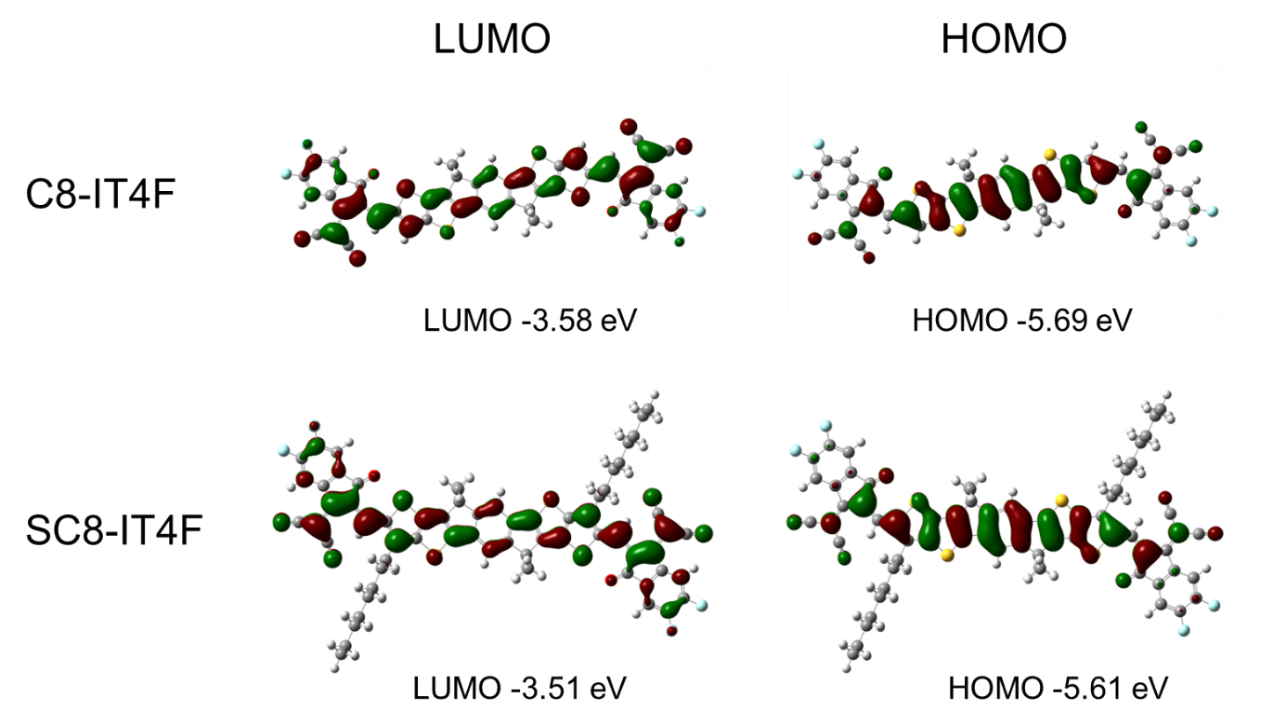


Figure S16. Frontier molecular orbitals of C8-IT4F and SC8-IT4F.


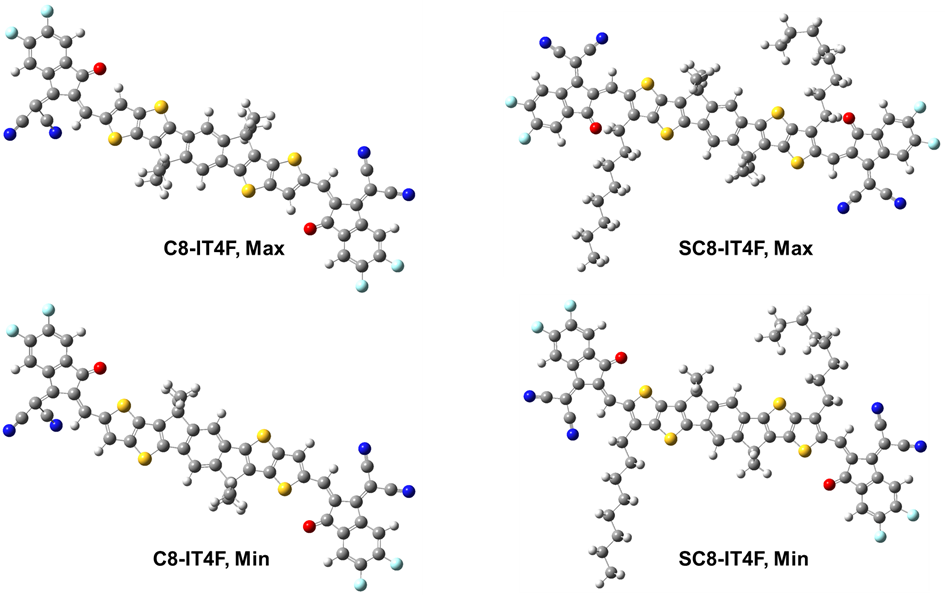


Figure S17. 3D structures of C8-IT4F and SC8-IT4F at maximum and minimum energy.


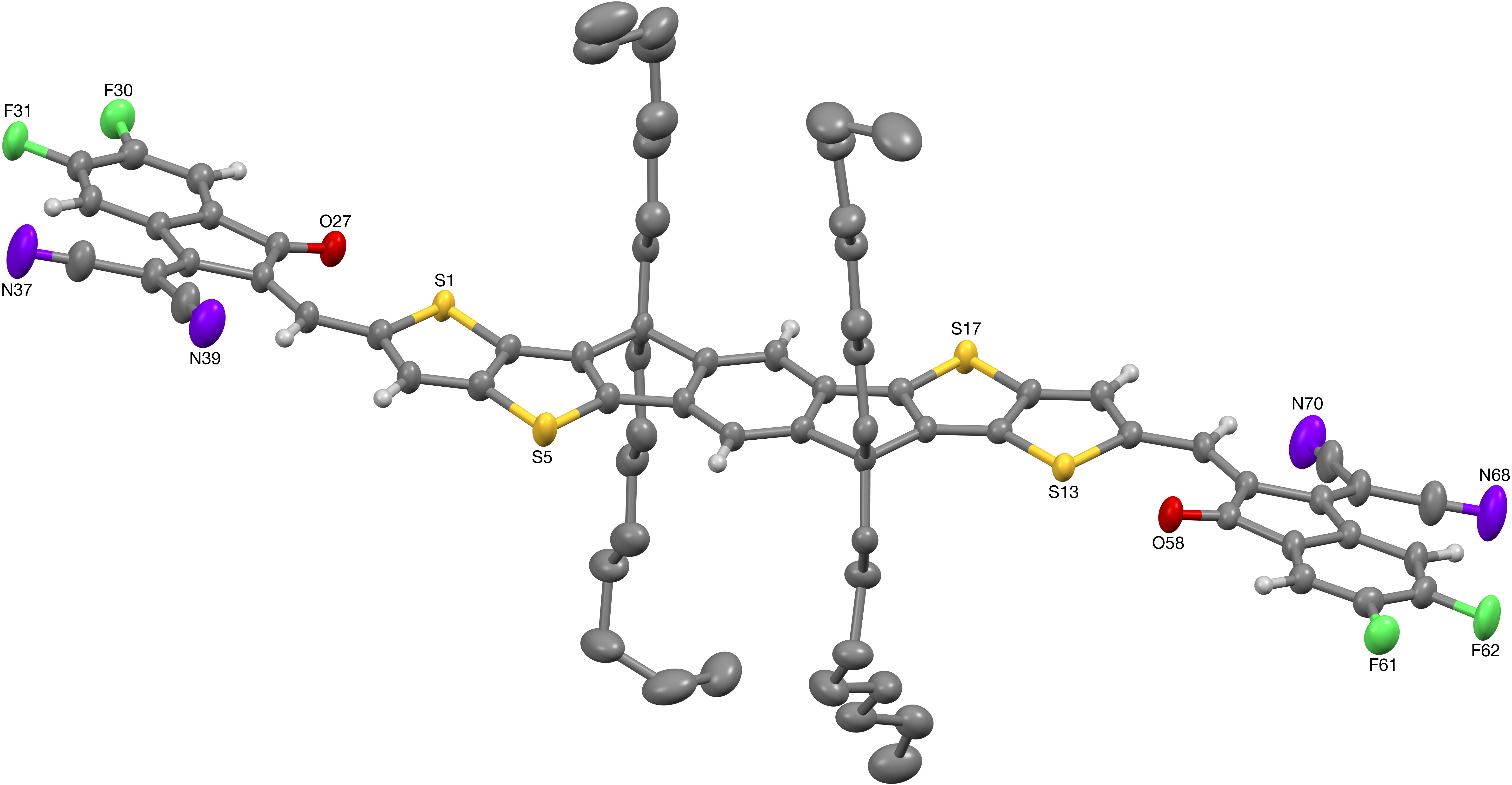


Figure S18. The crystal structure of C8-IT4F (50% probability ellipsoids).


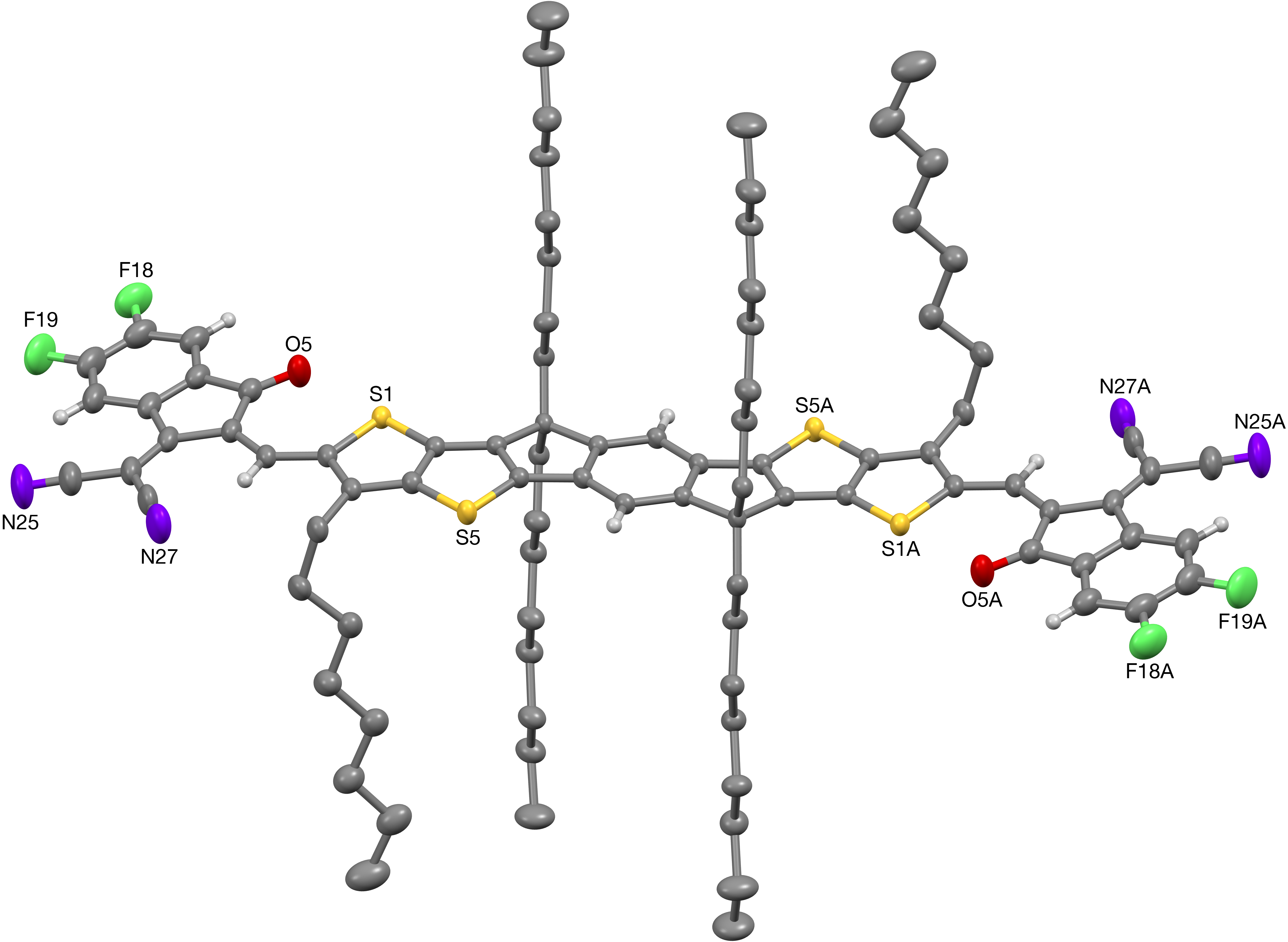


Figure S19. The crystal structure of the Ci-symmetric molecule SC8-IT4F (50% probability ellipsoids).


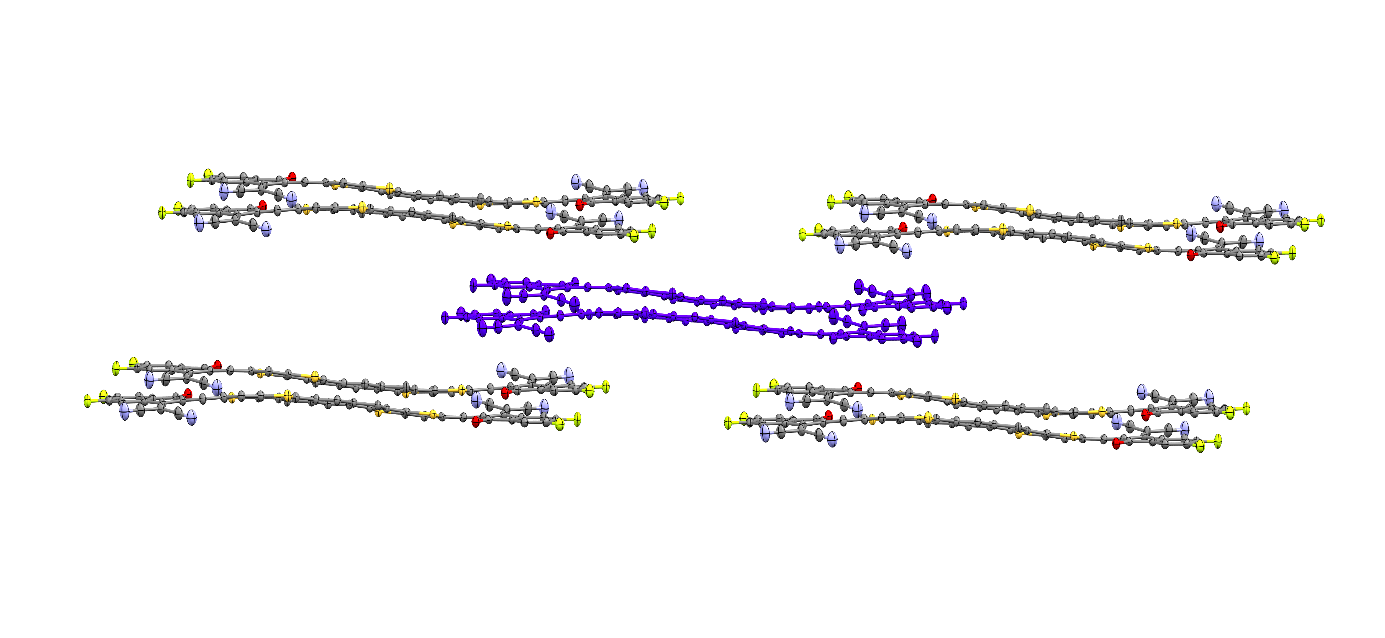


Figure S20. Crystal packing diagram of C8-IT4F.


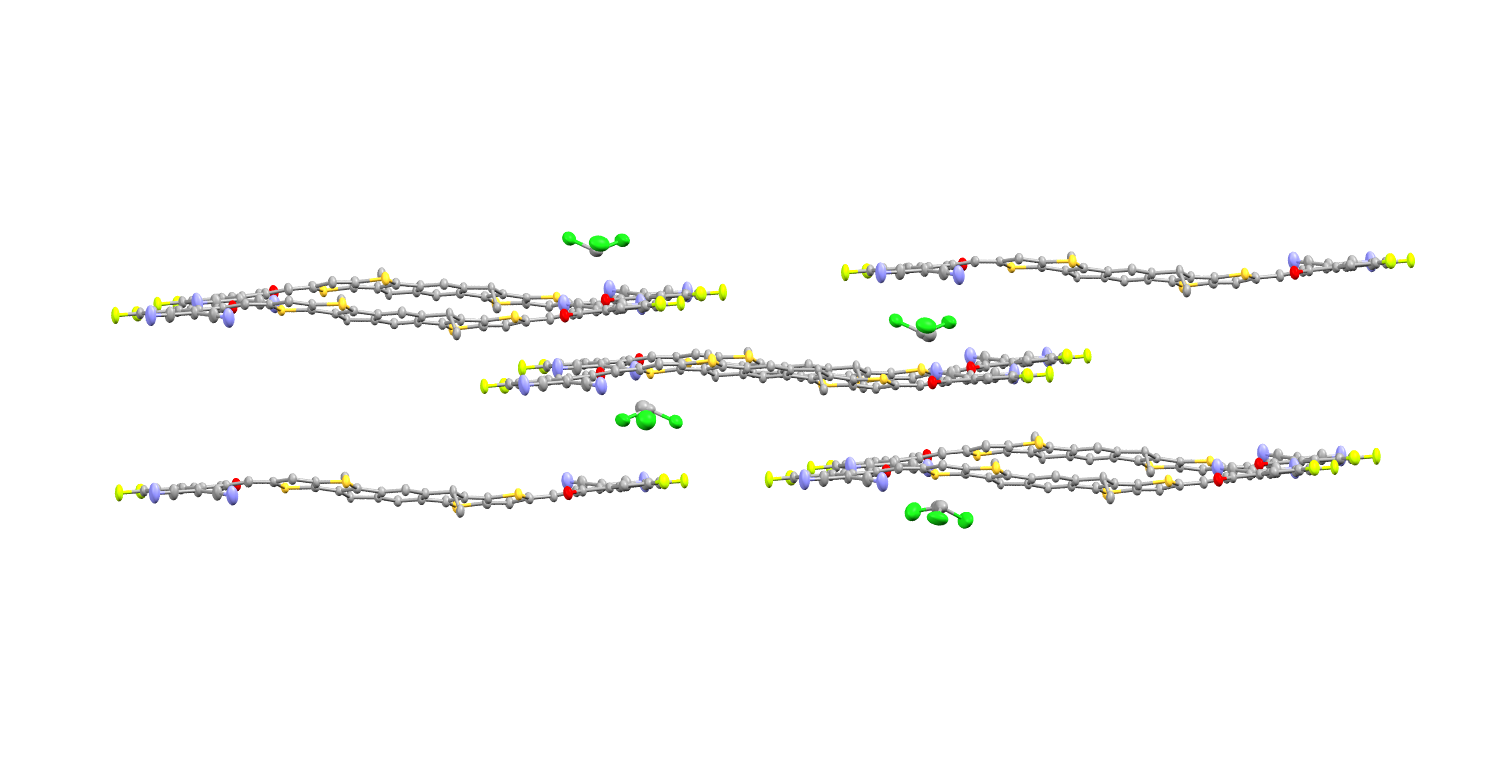


Figure S21. Crystal packing diagram of C8-IT4F with CHCl_3_ molecule.


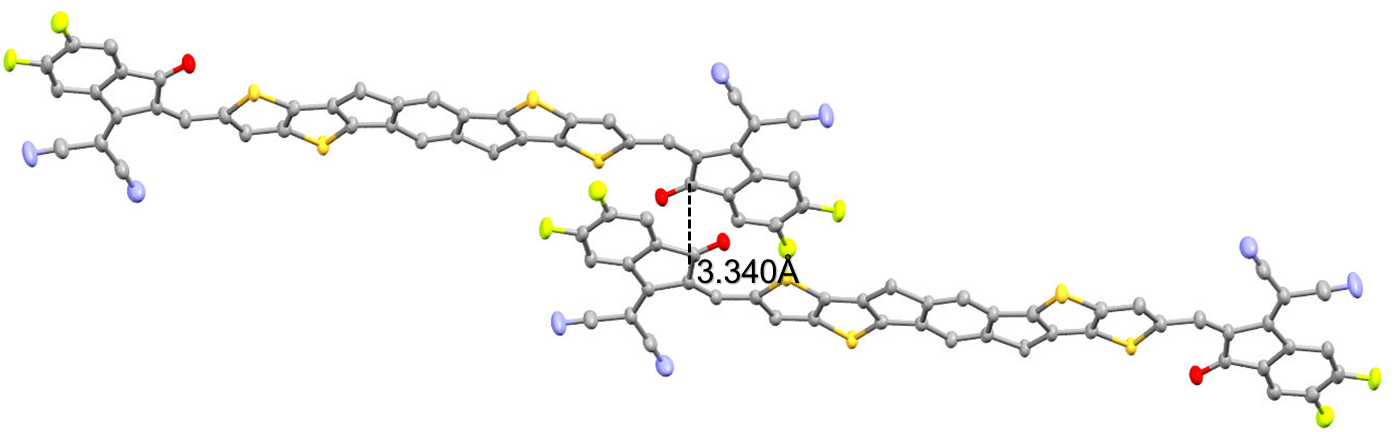


Figure S22. The distance between end groups of adjacent C8-IT4F molecules.

OSC Fabrication and Characterization

Organic photovoltaic devices were fabricated using PM6 as the donor and C8-IT4F or SC8-IT4F as the acceptor material (D:A ratio, 1:1.2). Donor concentration is 7.5 mg ml^–1^, dissolved in chloroform with 0.5% 1,8-diiodooctane (DIO). ITO/glass were pre-cleaned by sonication with soap, acetone, and isopropanol, respectively for 10 min before use. After drying, ITO substrates were exposed to UV ozone for 20 min. Then, the substrates were transferred to the N_2_ filled glove box for further deposition. 2PACz (0.5 mg/ml in absolute ethanol) were spin coated at 5000 rpm for 30 s. PM6:small molecule acceptors (SMAs) were mixed and stirred for ~1 h under 40 °C to dissolve the active materials. Then, active materials were spin coated at ~4000 rpm (film thickness ~110 nm) followed by drying under vacuum for 20 min. PDINN (2 mg/ml in methanol) as ETL were spin coated at 3000 rpm. Then, Ag (120 nm) were deposited using thermal evaporation under reduced pressure (< 3$\times$10^−6^ Pa). *J*-*V* curves were obtained by using Keithley 2400 source meter and Oriel Class 3A solar simulator calibrated using silicon reference from Newport. EQE spectra were measured using QE-R3011 (Enli tech). Both *J*-*V* and EQE were performed in N_2_ filled glove box. Light intensity dependence measurements were conducted by adjusting the light intensity using filter with different opacity. Thermal stability was measured by placing the cells in N_2_ glove box under dark conditions, then the PCE was periodically measured for every 24 h. Operational stability was measured by placing the cells in low humidity chamber and exposed to continuous LED light while applying the voltage to maintain the MPP (maximum power point) condition.

Mobility measurements

Space-charge-limited-current (SCLC) measurements were carried out under dark inside N_2_ filled glove box. The mobility ($\mu$) were determined by fitting the dark current using the equation:

Equation (S2)

$$J= \frac{9}{8}\varepsilon_{0}\varepsilon_{r}\mu\frac{V^{2}}{L^{3}}$$

*J* is the dark current. $\varepsilon_{0}$ and $\varepsilon_{r}$ are permittivity of vaccum and relative permittivity of materials, respectively. *V* is the effective voltage and *L* is the thickness of active materials. The mobility is calculated from the slope of *J^1^*^/2^-*V*.

Photo-CELIV were performed inside N_2_ glove box using PAIOS (Fluxim) (ramp rate 200 V ms^-1^, delay time 0 s, offset voltage 0 V, light pulse 100 $\mu$s). The mobility was determined using the equation:

$$\mu=\frac{2d^{2}}{3At_{max}^{2}}.\frac{1}{1+0.36\frac{j_{max}-j_{0}}{j_{0}}}$$

$d$ is the thickness, $A$ is the voltage ramp rate, *t*_max_ is the time required to reach the maximum current, $j_{0}$and $j_{max}$ are the initial current and maximum current, respectively.

The contact angle of DI water on neat thin films was measured using a Kruss DSA100. Images were analysed using Kruss Advance software with an automatic baseline and ellipse tangent fitting were used to extract the contact angle.

AFM samples were fabricated on clean glass substrates following the device active layer recipe. Images were obtained using a Bruker Dimension Icon AFM in tapping mode with a Tap150Al-G tip (Budget Sensors).


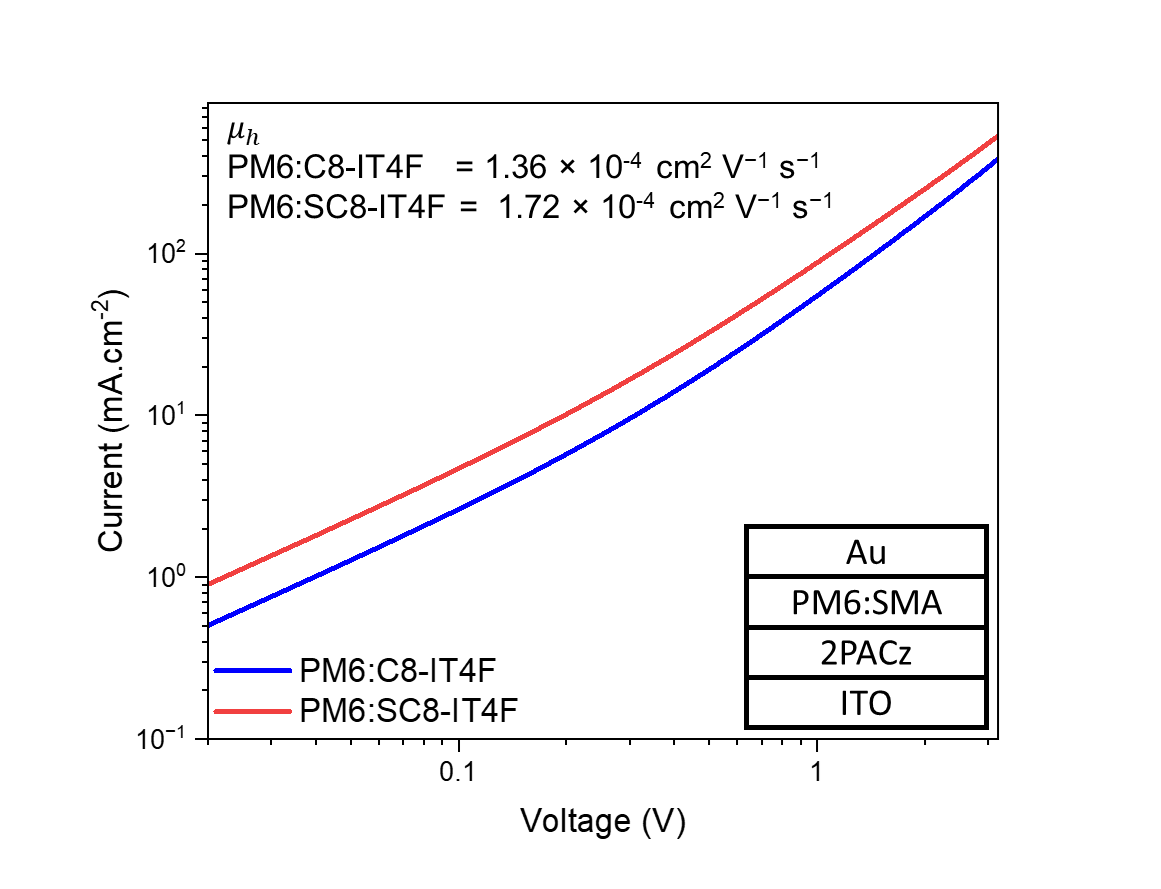


Figure S23. The hole mobilities (𝜇) of PM6:C8-IT4F and PM6:SC8-IT4F are 1.36 × 10^−4^ and 1.72 × 10^−4^ cm^2^ V^−1^ s^−1^.


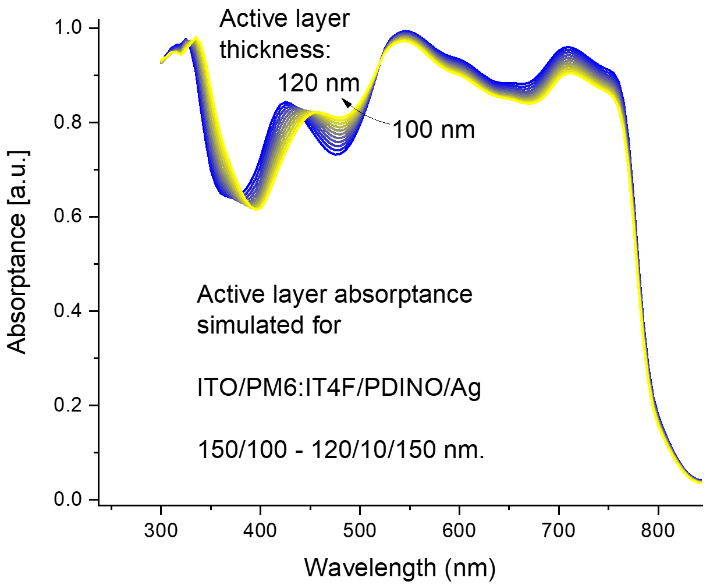


Figure S24. Optical simulation of an ITO/PM6:IT4F/PDINO/Ag sample. Note: for this qualitative estimation, we chose the most similar materials available in our ellipsometry-obtained library of refractive indexes^[4]^: PM6:IT4F instead of PM6/C8- or SC8-IT4F, PDINO instead of PDINN, and approximated as negligible the role of the very thin 2PACz layer. The transfer matrix based optical simulation was carried out with Setfos 5.4 from Fluxim.


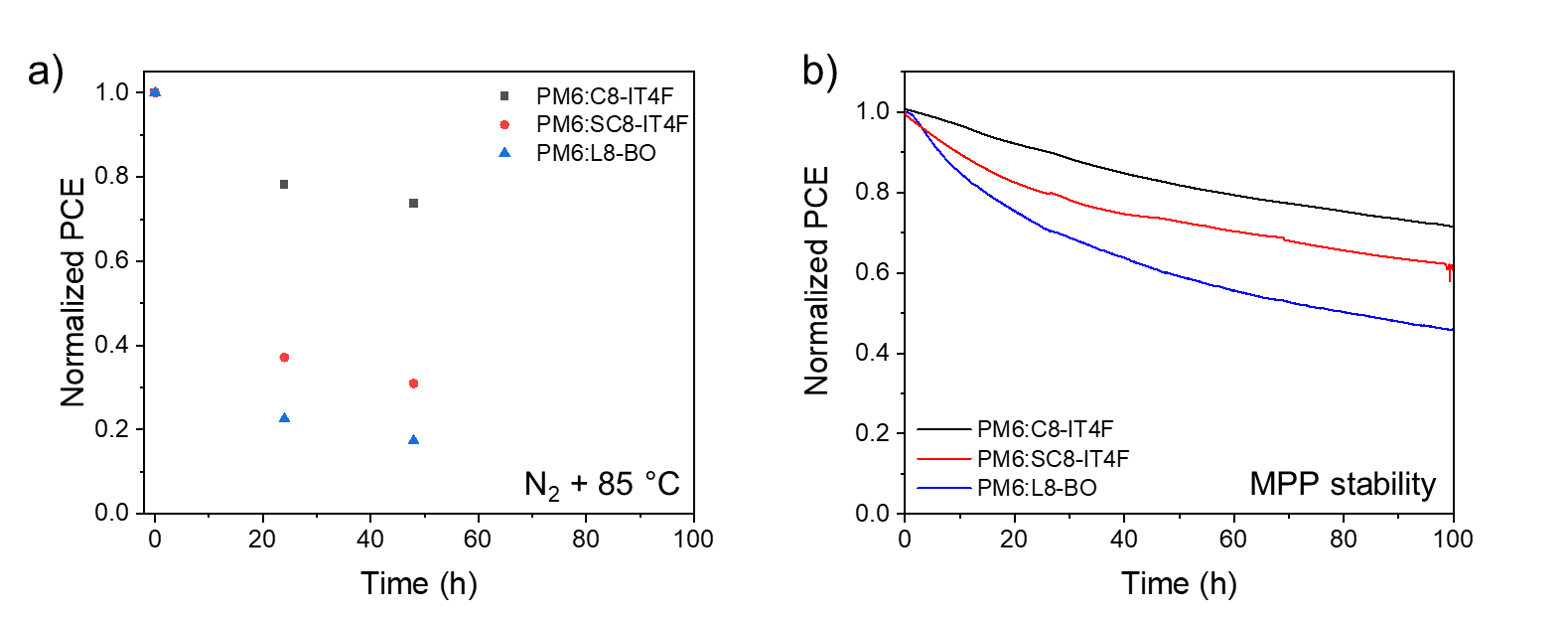


Figure S25. Stability test of the OSCs.


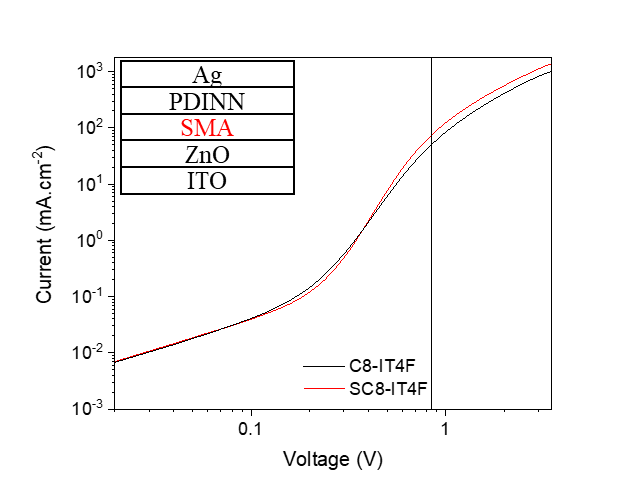


Figure S26. SCLC measurements performed on electron-only neat SMA. The electon mobilities (𝜇_𝑒_) of C8-IT4F and SC8-IT4F are 4.53 × 10^−4^ and 5.69 × 10^−4^ cm^2^ V^−1^ s^−1^.


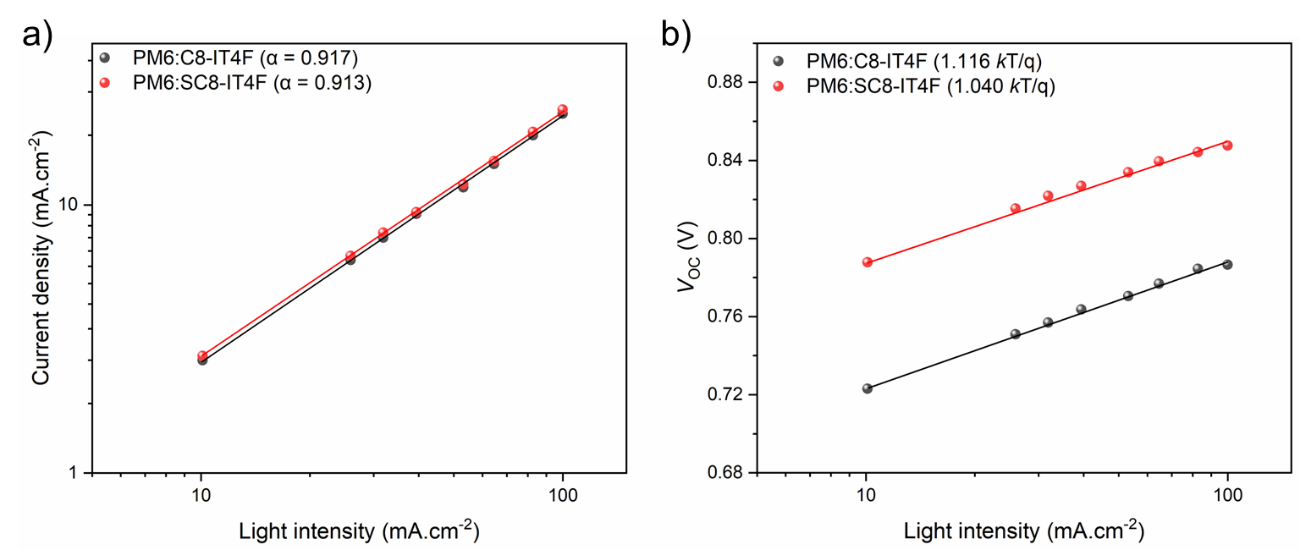


Figure S27. The dependence of J_SC_ and V_OC_ on light intensity of the optimized devices.

Photoluminescence emission spectroscopy (PL):

The steady-state photoluminescence (PL) emission spectra of the films spun on quartz were measured in air with a Jobin Yvon Fluorolog spectrofluorometer from Horiba. The samples were excited using Xe lamp source and detected using PMT-Si detector with an extended correction range up to 1000 nm. The films were excited at 690 nm. The emission spectra were corrected for the spectral sensitivity of the setup corresponding to the used configuration (detectors and gratings). Both samples were measured in both right angle configuration and at an angle of 60°.

Sensitive External Quantum Efficiency (sEQE) & Electroluminescence Spectroscopy (EL):

The EQE spectra with a high dynamic range (sensitive EQE) were acquired utilizing concentrated monochromatic light from a Xenon arc lamp paired with a monochromator under short-circuit conditions. An optical chopper modulated the light at 275 Hz. The device output current was demodulated for each incident photon energy using a Stanford Instruments SR 830 lock-in amplifier. Si photodiode was used to calibrate the light intensity.

The electroluminescence signal measurements were conducted on devices with an active area of 0.1 cm^2^ that were biased with DC current ranging between 1 - 20 mA (Keithley 2420). A collimator inside a nitrogen-filled glovebox captured the EL emission, which was then directed to a spectrograph (Princeton Instruments SP2300) via an optical fiber. A Ne/Ar and Mercury calibration light source was used to calibrate the system's wavelength (Princeton Instruments IntelliCal). Two different cameras were used to measure the signal over a range of wavelengths. A CCD camera (PIXIS, Princeton) was used to measure the visible range of 300 nm to 1100 nm, while a cryogenically cooled InGaAs detector (PyLoN® IR) was used to measure the NIR region with a spectral coverage of 800 nm to 2200 nm. The visible and infrared spectra were measured at center wavelengths of 900 nm and 1100 nm, respectively. To ensure accurate measurements, the spectral sensitivity of the setup was corrected using a tungsten halogen lamp (HL-3 plus, Ocean Optics) as a reference standard. The two signals were then combined by superimposing them. The visible signal was used when it was less noisy than the infrared signal, and the infrared signal was utilized when the visible signal became too noisy, and the emission was outside the detection range of the silicon detector.





Figure S28. Steady state PL of C8-IT4F and SC8-IT4F thin films.


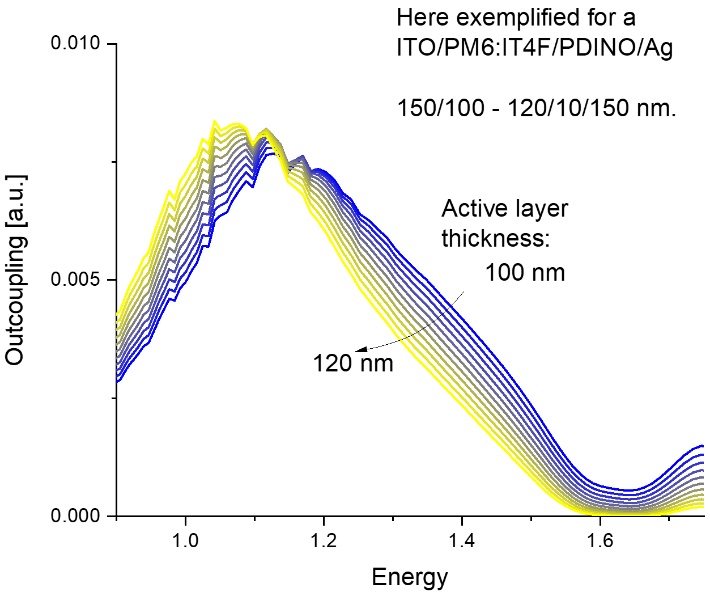


Figure S29. The effect of active layer thicknesses on the light outcoupling by stimulation.^[4]^ . The outcoupling figure represents the spectral shape light that would come out of a ITO/PM6:IT4F/PDINO/Ag layer stack, with the simple assumption of an emission profile centered in the center of the active layer, having a Gaussian shape, with a 50 nm Gaussian width, as simulated with Setfos 5.4 from Fluxim.





Figure S30. sEQE comparision of PM6:C8-IT4F and PM6:SC8-IT4F (left). The EQE_PV_ for both systems by using the optoelectronic reciprocity theorem (right). The sEQE not giving enough information especially for SMAs. What is more useful is using the optoelectronic reciprocity theorem (EQEpv) to get more order of magnitude therefore evaluate the energy losses.

Table S1. The radiative and non-radiative V_OC_ losses.

| System | *V*_OC_  (V) | *J*_SC_  (A cm^−2^) | *J*_0,rad_ | $\boldsymbol{V}_{\boldsymbol{OC}}^{\boldsymbol{rad}}$ | $\Delta\boldsymbol{V}_{\boldsymbol{OC}}^{\boldsymbol{nr}}=\boldsymbol{V}_{\boldsymbol{OC}}^{\boldsymbol{rad}}- \boldsymbol{V}_{\boldsymbol{OC}}$ |
| --- | --- | --- | --- | --- | --- |
| PM6:C8-IT4F | 0.8 | 219.2 | 2.04484E-18 | 1.176 | 0.376 |
| PM6:SC8-IT4F | 0.87 | 226.7 | 1.30548E-18 | 1.188 | 0.318 |

**Morphology characterisation**

Grazing-incidence wide-angle X-ray scattering (GIWAXS) measurements were conducted at Advanced Light Source (ALS), Lawrence Berkeley National Laboratory, Berkeley, CA at beamline 7.3.3. The samples were placed in a He-filled compartment during the measurements. Data were acquired at the critical angle (0.13°) of the film with a hard X-ray energy of 10 keV. The 2-D data were recorded with a Pilatus 2M detector with a pixel size of 172 um and placed at a distance of about 280 mm from the samples. The scattering data were analyzed using a custom Nika package for Igor Pro (Wavemetrics Inc.). The 1-D in-plane (IP) and out-of-plane (OoP) profiles from 10° cake sectors are shown in Figure S15.


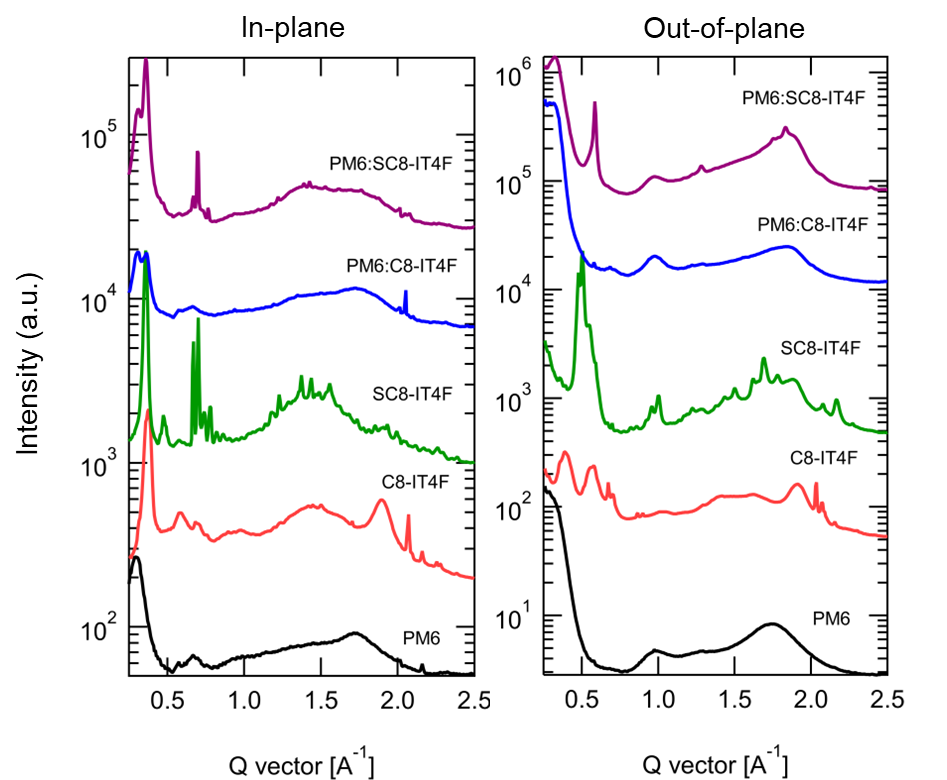


Figure S31. The 1-D in-plane (IP) and out-of-plane (OoP) profiles from 10° cake sectors from 2D data of neat and blend films.

A multipeak analysis of the structure in the region of the π-π and lamellar stacking peaks region of the 1-D out-of-plane profiles of the neat and blend systems by fitting Voigt peaks on a polynomial background. The peak fits to the π-π and lamellar peaks for the PM6:C8-IT4F and PM6:SC8-IT4F blends are shown in Figure S16. Besides the π-π stacking peak of the SMA at 1.83 Å^–1^, the other peaks seen in the PM6:SC8-IT4F data in that q-range likely arise from PM6 π-π stacking and/or higher order lamellar ordering peaks of the SC8-IT4F molecule. It may be noted here that XRD data indicates the ordering in both the SMAs might be non-orthorhombic and quite complex in nature. Therefore, a more complete analysis of the GIWAXS data of the two acceptor systems is beyond the scope of the current work. The Scherrer coherence lengths were calculated from all the fitted peaks using the Scherrer equation: CL = 2πK/Δq, where Δq is the full-width at half-maximum of the peak and K is a shape factor (K=1 was used here). The results from all the neat and blend samples are summarized in Table 3 and Table S2. Correlation between coherence length from acceptor first order lamellar peak and electron mobility is shown in Figure S17.


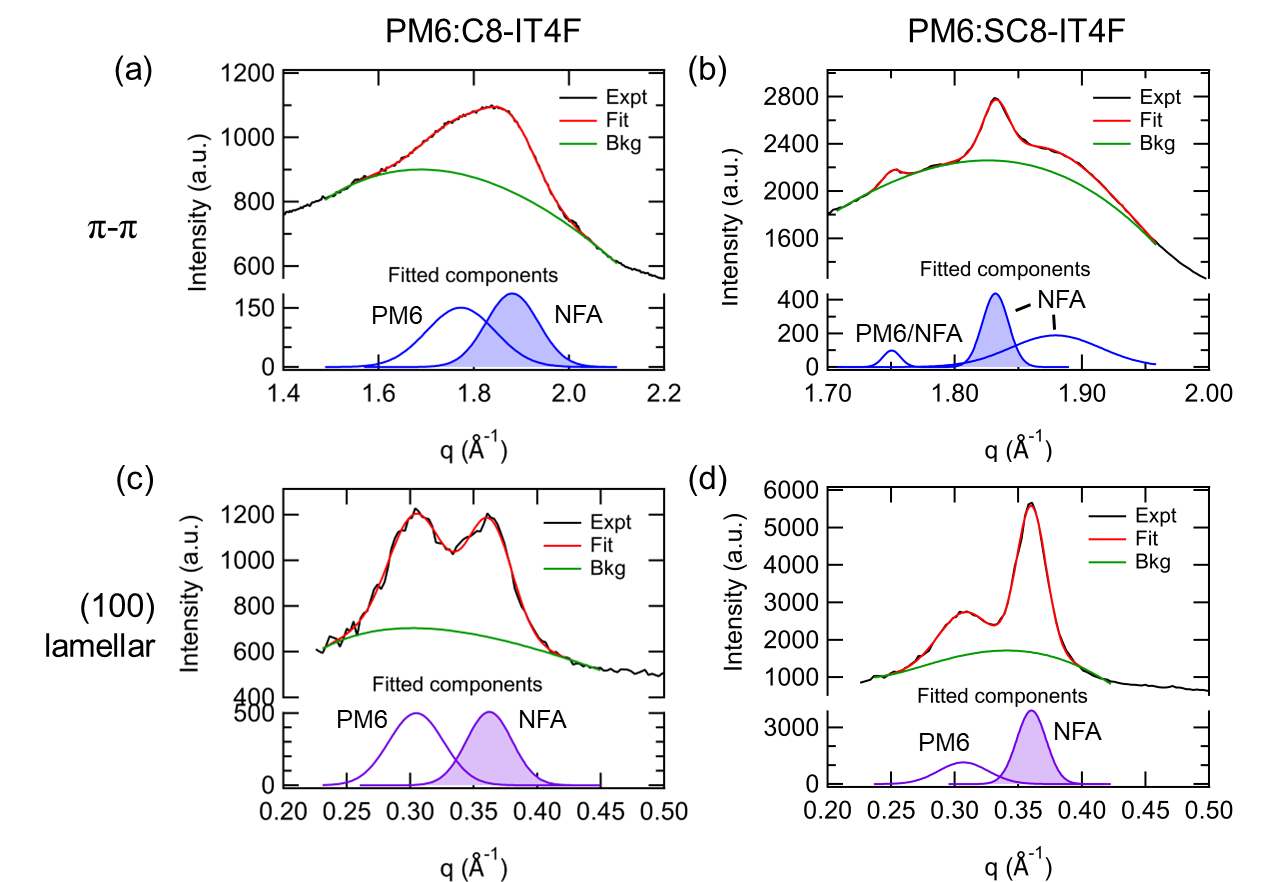


Figure S32. Multipeak fits to broad envelope covering π-π (top row) and (100) lamellar (bottom row) stacking peaks for PM6:C8-IT4F (left column) and PM6:SC8-IT4F (right column) blend films. The shaded areas indicate the SMA π-π and lamellar stacking peaks in the top and bottom rows, respectively.

GIWAXS pole figures were constructed from the missing wedge-corrected 2D data by integrating the intensities at each detector azimuth within the range of the peak of interest (lamellar or π-π stacking) of one or both the components in the film. A linear background defined by the intensities at the two ends of the integrated q-range was subtracted. A geometry correction was applied to the pole figures by multiplying the raw intensity at each azimuthal angle by the sine of the detector azimuth. The detector azimuth (χ) is defined as the angle of the observed diffraction from the normal to the substrate. The relative degree of crystallinity (rDoC) was calculated by integrating the geometry corrected intensities over the detector azimuth, rDoC = $\int_{0}^{\pi/2} I(\chi)\sin\chi d\chi$, and normalized by the illuminated volume (sample length along the beam and film thickness). Assuming similar structure factors the normalized rDoC calculated from the pole figure in the q-range 0.27–0.4 Å^−1^ covering the polymer and acceptor lamellar peaks for the PM6:SC8-IT4F blend was found to be about double that of the PM6:C8-IT4F blend (1.00 vs 0.47).

Table S2. Spacings and coherence lengths for the acceptor IP (100) lamellar peak in GIWAXS data of C8-IT4F and SC8-IT4F neat and blend films, OoP π-π and IP (100) lamellar peaks of PM6 neat sample. Coherence length uncertainty values were calculated from error in FWHM in the fits and given in parenthesis.

| Sample | Peak type | q location (Å^-1^) | Spacing (Å) | Coherence Length (nm) |
| --- | --- | --- | --- | --- |
| C8-IT4F neat | (100) | 0.37 | 16.83 | 14.9 (0.4) |
| SC8-IT4F neat | (100) | 0.36 | 17.44 | 29.3 (0.4) |
| PM6:C8-IT4F | (100) SMA | 0.36 | 17.35 | 14.8 (0.6) |
| PM6:SC8-IT4F | (100) SMA | 0.36 | 17.44 | 23.1 (0.3) |
| PM6 neat | π-π | 1.74 | 3.61 | 2.4 (0.1) |
|  | (100) | 0.30 | 21.12 | 6.9 (0.2) |


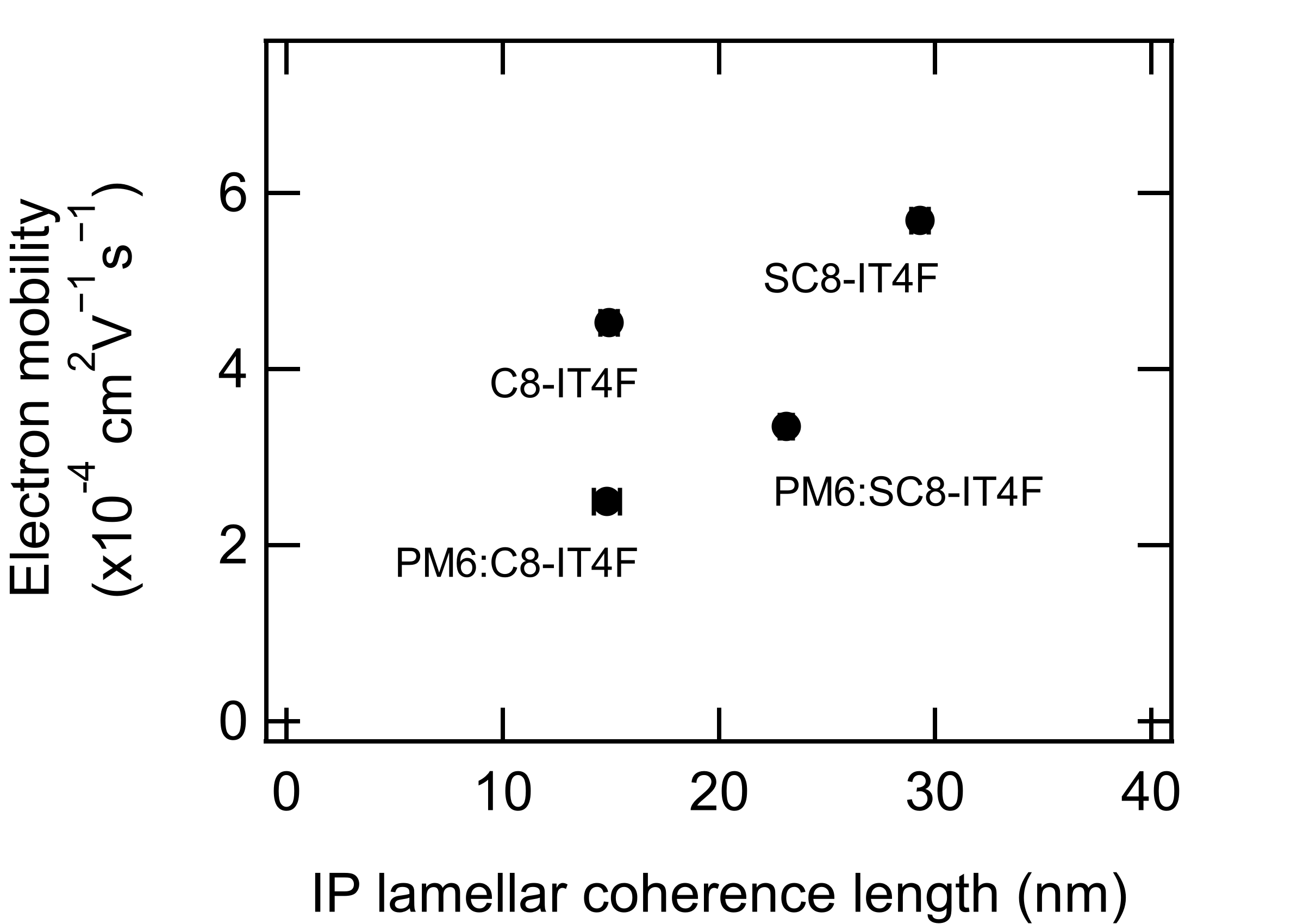


Figure S33. Plot of electron mobility against coherence length of IP (100) stacking peak of the acceptor in C8-IT4F and SC8-IT4F neat and blend films.


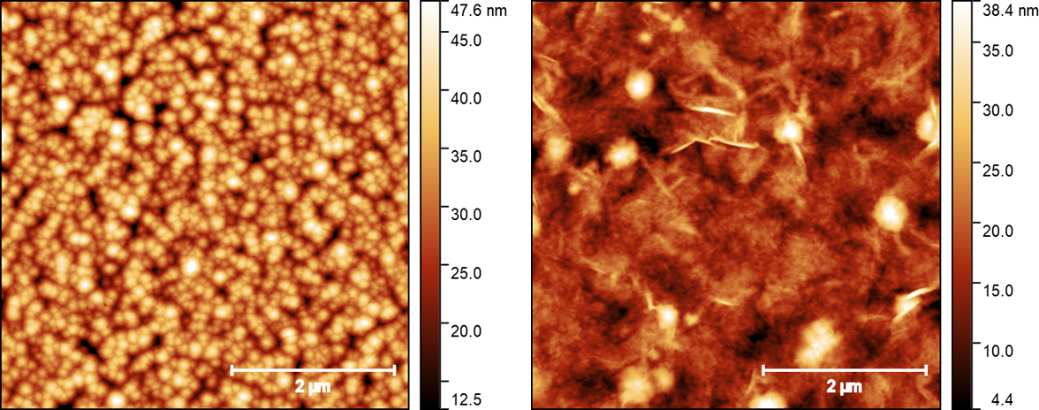


Figure S34. AFM images of the C8-IT4F (left, roughness: 5.6 nm) and SC8-IT4F (right, roughness: 4.6 nm) neat films. The two acceptors exhibit different crystallinity, with larger more fibril domains in SC8-IT4F, but more consistent small domains in C8-IT4F.


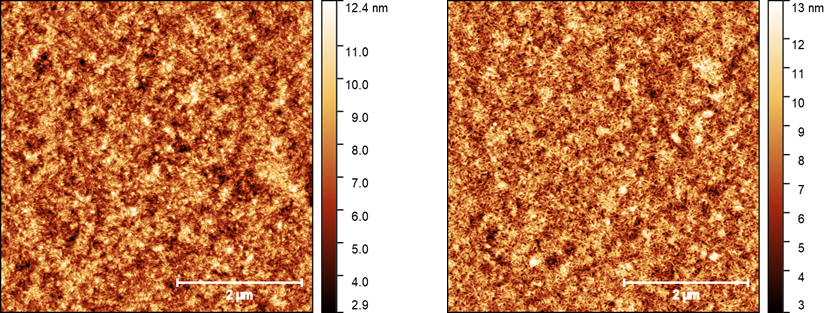


Figure S35. AFM images of the PM6:C8-IT4F (left, roughness: 2.7 nm) and PM6:SC8-IT4F (right, roughness: 4.6 nm) blend films. Small differences between their blends.

Table S3. Summary of photovoltaic parameters of non-Y acceptor based OPV cells.

| Donor:Acceptor | *V*_OC_  [V] | *J*_SC_  [mA cm^-2^] | FF  [%] | PCE  [%] | Ref. |
| --- | --- | --- | --- | --- | --- |
| PTB7-Th: ITIC | 0.81 | 14.21 | 59.1 | 6.80 | ^[5]^ |
| PDBT-T1:6a | 1.00 | 12.99 | 71.5 | 9.28 | ^[6]^ |
| PTB7-Th:ATT-1 | 0.87 | 16.48 | 70 | 10.07 | ^[7]^ |
| J71:ITIC | 0.94 | 17.32 | 69.77 | 11.41 | ^[8]^ |
| PBDB-T:IT-M | 0.94 | 17.44 | 73.5 | 12.05 | ^[9]^ |
| PTzBI:N2200 | 0.85 | 15.17 | 70.36 | 9.16 | ^[10]^ |
| PBDB-T:NFBDT | 0.868 | 17.85 | 67.2 | 10.42 | ^[11]^ |
| FTAZ:INIC3 | 0.857 | 19.44 | 67.4 | 11.5 | ^[12]^ |
| PBDB-T-SF:IT-4F | 0.88 | 20.88 | 71.3 | 13.1 | ^[13]^ |
| PTB7-Th:SiOTIC-4F | 0.65 | 21.6 | 61.4 | 9.0 | ^[14]^ |
| PBDB-T:DF-PCIC | 0.91 | 15.66 | 72 | 10.14 | ^[15]^ |
| H22:IDIC | 0.942 | 15.38 | 71.15 | 10.29 | ^[16]^ |
| PBDB–TF:HF–PCIC | 0.91 | 11.78 | 70.7 | 11.49 | ^[17]^ |
| PBDB-T:ITC6-IC | 0.97 | 16.41 | 73 | 11.61 | ^[18]^ |
| PBDB-TF:HC-PCIC | 0.89 | 18.13 | 72.06 | 11.75 | ^[19]^ |
| PBDB-T:DOC2C6-2F | 0.85 | 21.35 | 73.15 | 13.24 | ^[20]^ |
| PBDB-TF:PTIC | 0.93 | 16.73 | 66 | 10.27 | ^[21]^ |
| PBDB-T:o-4TBC-2F | 0.76 | 20.48 | 65.7 | 10.26 | ^[22]^ |
| PBDB-T:BTOR-IC4F | 0.80 | 20.57 | 69.6 | 11.48 | ^[23]^ |
| PM7:BT2FIDT-4Cl | 0.97 | 18.1 | 71.5 | 12.5 | ^[24]^ |
| PBDB-TF:PTB4Cl | 0.93 | 19.01 | 72.17 | 12.76 | ^[25]^ |
| PM6:BDC-4F-C8 | 0.895 | 21.32 | 65.6 | 12.53 | ^[26]^ |
| PM6:TPDC-4F | 0.852 | 22.19 | 70.6 | 13.35 | ^[27]^ |
| J52:BN-2F | 0.813 | 25.25 | 70.78 | 14.53 | ^[28]^ |
| J52:NoCA-5 | 0.814 | 26.02 | 69.96 | 14.82 | ^[29]^ |
| PBDB-TF:A4T-16 | 0.876 | 21.8 | 79.8 | 15.2 | ^[30]^ |
| D18: 2BTh-2F(C2) | 0.90 | 23.61 | 72.30 | 15.44 | ^[31]^ |
| PBDB-T:DBT-HD | 0.85 | 21.75 | 73.39 | 13.57 | ^[32]^ |
| PM6:4TC-4F-C8C8 | 0.93 | 21.01 | 0.76 | 14.85 | ^[33]^ |
| PM6:WA1 | 0.86 | 22.65 | 79.31 | 15.45 | ^[34]^ |
| D18:TTS-2F | 0.83 | 24.23 | 76.10 | 15.29 | ^[35]^ |
| ID-C6Ph-ST-4F | 0.88 | 23.19 | 75.27 | 15.36 | ^[36]^ |
| PM6:ThPy6 | 0.871 | 23.43 | 78.9 | 16.11 | ^[37]^ |
| D18:TTPIC-4F | 0.883 | 25.24 | 76.6 | 17.1 | ^[38]^ |
| D18: IDTT-SiO-IC | 1.018 | 15.49 | 72.75 | 11.47 | ^[39]^ |
| D18:3T-2 | 0.87 | 17.85 | 71.21 | 11.12 | ^[40]^ |
| D18:o-AT-2Cl | 0.914 | 20.1 | 69.6 | 12.8 | ^[41]^ |
| D18:ZITI-N-4F | 0.945 | 20.85 | 63.31 | 12.87 | ^[42]^ |
| PB2: TBT-2 | 0.873 | 21.16 | 71.7 | 13.25 | ^[43]^ |
| PM6: Ph-BDD | 0.86 | 24.07 | 65 | 13.64 | ^[44]^ |
| PM6:FEH2C8-2Cl | 0.918 | 20.12 | 79.04 | 14.6 | ^[45]^ |
| PM6:M36 | 0.91 | 23.93 | 78.4 | 17 | ^[46]^ |

**Reference**

[1] Z. Fei, F. D. Eisner, X. Jiao, M. Azzouzi, J. A. Rohr, Y. Han, M. Shahid, A. S. R. Chesman, C. D. Easton, C. R. McNeill, T. D. Anthopoulos, J. Nelson, M. Heeney, *Adv. Mater.* **2018**, 30, 1705209.

[2] S. E. Root, M. A. Alkhadra, D. Rodriquez, A. D. Printz, D. J. Lipomi, *Chem. Mater.* **2017**, 29, 2646.

[3] a)G. M. Sheldrick, *Acta Crystallogr. C* **2015**, C71, 3; b)O. V. Dolomanov, L. J. Bourhis, R. J. Gildea, J. A. K. Howard, H. Puschmann, *J. Appl. Cryst.* **2009**, 42, 339; c)B. A. SHELXTL v5.1, Madison, WI, **1998**.

[4] S. Karuthedath, J. Gorenflot, Y. Firdaus, N. Chaturvedi, C. S. P. De Castro, G. T. Harrison, J. I. Khan, A. Markina, A. H. Balawi, T. A. D. Peña, W. Liu, R.-Z. Liang, A. Sharma, S. H. K. Paleti, W. Zhang, Y. Lin, E. Alarousu, S. Lopatin, D. H. Anjum, P. M. Beaujuge, S. De Wolf, I. McCulloch, T. D. Anthopoulos, D. Baran, D. Andrienko, F. Laquai, *Nat. Mater.* **2021**, 20, 378.

[5] Y. Lin, J. Wang, Z.-G. Zhang, H. Bai, Y. Li, D. Zhu, X. Zhan, *Adv. Mater.* **2015**, 27, 1170.

[6] D. Meng, H. Fu, C. Xiao, X. Meng, T. Winands, W. Ma, W. Wei, B. Fan, L. Huo, N. L. Doltsinis, Y. Li, Y. Sun, Z. Wang, *J. Am. Chem. Soc.* **2016**, 138, 10184.

[7] F. Liu, Z. Zhou, C. Zhang, T. Vergote, H. Fan, F. Liu, X. Zhu, *J. Am. Chem. Soc.* **2016**, 138, 15523.

[8] H. Bin, L. Gao, Z.-G. Zhang, Y. Yang, Y. Zhang, C. Zhang, S. Chen, L. Xue, C. Yang, M. Xiao, Y. Li, *Nat. Commun.* **2016**, 7, 13651.

[9] S. Li, L. Ye, W. Zhao, S. Zhang, S. Mukherjee, H. Ade, J. Hou, *Adv. Mater.* **2016**, 28, 9423.

[10] B. Fan, L. Ying, Z. Wang, B. He, X.-F. Jiang, F. Huang, Y. Cao, *Energy Environ. Sci.* **2017**, 10, 1243.

[11] B. Kan, H. Feng, X. Wan, F. Liu, X. Ke, Y. Wang, Y. Wang, H. Zhang, C. Li, J. Hou, Y. Chen, *J. Am. Chem. Soc.* **2017**, 139, 4929.

[12] S. Dai, F. Zhao, Q. Zhang, T.-K. Lau, T. Li, K. Liu, Q. Ling, C. Wang, X. Lu, W. You, X. Zhan, *J. Am. Chem. Soc.* **2017**, 139, 1336.

[13] W. Zhao, S. Li, H. Yao, S. Zhang, Y. Zhang, B. Yang, J. Hou, *J. Am. Chem. Soc.* **2017**, 139, 7148.

[14] J. Lee, S.-J. Ko, M. Seifrid, H. Lee, B. R. Luginbuhl, A. Karki, M. Ford, K. Rosenthal, K. Cho, T.-Q. Nguyen, G. C. Bazan, *Adv. Energy Mater.* **2018**, 8, 1801212.

[15] S. Li, L. Zhan, F. Liu, J. Ren, M. Shi, C.-Z. Li, T. P. Russell, H. Chen, *Adv. Mater.* **2018**, 30, 1705208.

[16] H. Bin, J. Yao, Y. Yang, I. Angunawela, C. Sun, L. Gao, L. Ye, B. Qiu, L. Xue, C. Zhu, C. Yang, Z.-G. Zhang, H. Ade, Y. Li, *Adv. Mater.* **2018**, 30, 1706361.

[17] S. Li, L. Zhan, W. Zhao, S. Zhang, B. Ali, Z. Fu, T.-K. Lau, X. Lu, M. Shi, C.-Z. Li, J. Hou, H. Chen, *J. Mater. Chem. A* **2018**, 6, 12132.

[18] Z. Zhang, J. Yu, X. Yin, Z. Hu, Y. Jiang, J. Sun, J. Zhou, F. Zhang, T. P. Russell, F. Liu, W. Tang, *Adv. Funct. Mater.* **2018**, 28, 1705095.

[19] S. Li, L. Zhan, C. Sun, H. Zhu, G. Zhou, W. Yang, M. Shi, C.-Z. Li, J. Hou, Y. Li, H. Chen, *J. Am. Chem. Soc.* **2019**, 141, 3073.

[20] H. Huang, Q. Guo, S. Feng, C. e. Zhang, Z. Bi, W. Xue, J. Yang, J. Song, C. Li, X. Xu, Z. Tang, W. Ma, Z. Bo, *Nat. Commun.* **2019**, 10, 3038.

[21] Z.-P. Yu, Z.-X. Liu, F.-X. Chen, R. Qin, T.-K. Lau, J.-L. Yin, X. Kong, X. Lu, M. Shi, C.-Z. Li, H. Chen, *Nat. Commun.* **2019**, 10, 2152.

[22] Y.-N. Chen, M. Li, Y. Wang, J. Wang, M. Zhang, Y. Zhou, J. Yang, Y. Liu, F. Liu, Z. Tang, Q. Bao, Z. Bo, *Angew. Chem. Int. Ed.* **2020**, 59, 22714.

[23] Y. Wang, Z. Liu, X. Cui, C. Wang, H. Lu, Y. Liu, Z. Fei, Z. Ma, Z. Bo, *J. Mater. Chem. A* **2020**, 8, 12495.

[24] C. Zhang, X. Song, K.-K. Liu, M. Zhang, J. Qu, C. Yang, G.-Z. Yuan, A. Mahmood, F. Liu, F. He, D. Baran, J.-L. Wang, *Small* **2020**, 16, 1907681.

[25] T.-J. Wen, Z.-X. Liu, Z. Chen, J. Zhou, Z. Shen, Y. Xiao, X. Lu, Z. Xie, H. Zhu, C.-Z. Li, H. Chen, *Angew. Chem. Int. Ed.* **2021**, 60, 12964.

[26] D. Luo, X. Lai, N. Zheng, C. Duan, Z. Wang, K. Wang, A. K. K. Kyaw, *Chem. Eng. J.* **2021**, 420, 129768.

[27] D. Luo, L. Li, Y. Shi, J. Zhang, K. Wang, X. Guo, A. K. K. Kyaw, *J. Mater. Chem. A* **2021**, 9, 14948.

[28] X. Zhang, L. Qin, J. Yu, Y. Li, Y. Wei, X. Liu, X. Lu, F. Gao, H. Huang, *Angew. Chem. Int. Ed.* **2021**, 60, 12475.

[29] X. Zhang, C. Li, L. Qin, H. Chen, J. Yu, Y. Wei, X. Liu, J. Zhang, Z. Wei, F. Gao, Q. Peng, H. Huang, *Angew. Chem. Int. Ed.* **2021**, 60, 17720.

[30] L. Ma, S. Zhang, J. Zhu, J. Wang, J. Ren, J. Zhang, J. Hou, *Nat. Commun.* **2021**, 12, 5093.

[31] X. Wang, H. Lu, Y. Liu, A. Zhang, N. Yu, H. Wang, S. Li, Y. Zhou, X. Xu, Z. Tang, Z. Bo, *Adv. Energy Mater.* **2021**, 11, 2102591.

[32] J. Cao, H. Wang, L. Yang, F. Du, J. Yu, W. Tang, *Chem. Eng. J.* **2022**, 427, 131828.

[33] J. Ouyang, F. Wu, X. Zhao, X. Yang, *Small* **2022**, 18, 2201769.

[34] P. Wang, Y. Li, C. Han, J. Wang, F. Bi, N. Zheng, J. Yang, J. Wang, X. Bao, *J. Mater. Chem. A* **2022**, 10, 17808.

[35] X. Zheng, W. Liu, H. Wang, X. Man, G. Ran, X. Yu, H. Lu, Z. Bi, Y. Liu, A. Zhang, W. Ma, X. Xu, Z. Tang, W. Zhang, Z. Bo, *Cell Rep. Phys. Sci.* **2022**, 3, 101169.

[36] P. Wang, F. Bi, Y. Li, C. Han, N. Zheng, S. Zhang, J. Wang, Y. Wu, X. Bao, *Adv. Funct. Mater.* **2022**, 32, 2200166.

[37] Z. Luo, T. Liu, J. Oh, R. Ma, J. Miao, F. Ni, G. Zhang, R. Sun, C. e. Zhang, Z. Chen, Y. Zou, J. Min, C. Yang, H. Yan, C. Yang, *Adv. Funct. Mater.* **2022**, 32, 2203200.

[38] J. Zhou, D. He, Y. Li, F. Huang, J. Zhang, C. Zhang, Y. Yuan, Y. Lin, C. Wang, F. Zhao, *Adv. Mater.* **2022**, 35, e2207336.

[39] F. Meng, Y. Qin, Y. Zheng, Z. Zhao, Y. Sun, Y. Yang, K. Gao, D. Zhao, *Angew. Chem. Int. Ed.* **2023**, 62, e202217173.

[40] Y. Zhou, P. Liu, S. Shen, M. Li, R. Qin, X. Tang, C. Qin, J. Song, Z. Bo, L. Zhang, *J. Mater. Chem. A* **2023**, 11, 7498.

[41] R. Zheng, C. Zhang, A. Zhang, J. Xue, X. Xu, Y. Liu, C. J. Su, W. Ma, C. Yang, Z. Bo, *ACS Appl. Mater. Interfaces* **2023**, 15, 4275.

[42] Y. Jiang, W. Su, W. Wang, F. Liu, W. Liu, S. Xu, W. Zhang, J. Hou, X. Zhu, *Fundam. Res.* **2023**, <https://doi.org/10.1016/j.fmre.2023.03.010>.

[43] N. Yang, T. Zhang, S. Wang, C. An, S. Seibt, G. Wang, J. Wang, Y. Yang, W. Wang, Y. Xiao, H. Yao, S. Zhang, W. Ma, J. Hou, *Small Methods* **2023**, 10.1002/smtd.202300036, e2300036.

[44] S. Deng, W. Luo, L. Zhang, G. Xie, S. Lei, M. Luo, Z. Wu, D. Yuan, J. Liang, Z. Xie, J. Chen, *J. Mater. Chem. A* **2023**, 11, 3437.

[45] Z. Li, B. Zhou, S. Zhang, C. Jiang, Y. Zou, S. Li, Y. Yang, Z. Yao, X. Wan, Y. Chen, *J. Mater. Chem. A* **2023**, 11, 700.

[46] Y. Ma, R. Sun, Z. Chen, S. Zhang, D. Cai, S. Wan, W. Lin, S.-Q. Zhang, Q. Tu, W. Ma, J. Min, X. Hao, Q. Zheng, *Nano Energy* **2023**, 107, 108116.
